# Cost‐effectiveness of adjuvant chemotherapy for high‐risk stage II and stage III colon cancer in South Africa

**DOI:** 10.1002/cam4.6199

**Published:** 2023-06-15

**Authors:** Sarah Xinhui Tan, Yoanna Pumpalova, Alexandra M. Rogers, Kishan Bhatt, Candice‐lee Herbst, Paul Ruff, Alfred I. Neugut, Chin Hur

**Affiliations:** ^1^ Department of Medicine, Vagelos College of Physicians and Surgeons Columbia University New York New York USA; ^2^ Department of Internal Medicine, Faculty of Health Sciences University of the Witwatersrand Johannesburg South Africa; ^3^ Noncommunicable Diseases Research Division, Wits Health Consortium (PTY) Ltd Johannesburg South Africa; ^4^ SAMRC/Wits Developmental Pathways to Health Research Unit, Department of Paediatrics, Faculty of the Health Sciences University of the Witwatersrand Johannesburg South Africa; ^5^ Division of Medical Oncology, Department of Medicine, Faculty of Health Sciences University of the Witwatersrand Johannesburg South Africa; ^6^ Herbert Irving Comprehensive Cancer Center, Vagelos College of Physicians and Surgeons Columbia University New York New York USA; ^7^ Department of Epidemiology, Mailman School of Public Health Columbia University New York New York USA

**Keywords:** adjuvant chemotherapy, cancer treatment, colon cancer, cost‐effectiveness, South Africa

## Abstract

**Background:**

Colon cancer incidence is rising in low‐ and middle‐income countries (LMICs), where resource limitations and cost often dictate treatment decisions. In this study, we evaluate the cost‐effectiveness of adjuvant chemotherapy for high‐risk stage II and stage III colon cancer treatment in South Africa (ZA) and illustrate how such analyses can inform cancer treatment recommendations in a LMIC.

**Methods:**

We created a decision‐analytic Markov model to compare lifetime costs and outcomes for patients with high‐risk stage II and stage III colon cancer treated with three adjuvant chemotherapy regimens in a public hospital in ZA: capecitabine and oxaliplatin (CAPOX) for 3 and 6 months, and capecitabine for 6 months, compared to no adjuvant treatment. The primary outcome was the incremental cost‐effectiveness ratio (ICER) in international dollars (I$) per disability‐adjusted life‐year (DALY) averted, at a willingness‐to‐pay (WTP) threshold equal to the 2021 ZA gross domestic product per capita (I$13,764/DALY averted).

**Results:**

CAPOX for 3 months was cost‐effective for both patients with high‐risk stage II and patients with stage III colon cancer (ICER = I$250/DALY averted and I$1042/DALY averted, respectively), compared to no adjuvant chemotherapy. In subgroup analyses of patients by tumor stage and number of positive lymph nodes, for patients with high‐risk stage II colon cancer and T4 tumors, and patients with stage III colon cancer with T4 or N2 disease. CAPOX for 6 months was cost‐effective and the optimal strategy. The optimal strategy in other settings will vary by local WTP thresholds. Decision analytic tools can be used to identify cost‐effective cancer treatment strategies in resource‐constrained settings.

**Conclusion:**

Colon cancer incidence is increasing in low‐ and middle‐income countries, including South Africa, where resource constraints can impact treatment decisions. This cost‐effectiveness study evaluates three systemic adjuvant chemotherapy options, compared to surgery alone, for patients in South African public hospitals after surgical resection for high‐risk stage II and stage III colon cancer. Doublet adjuvant chemotherapy (capecitabine and oxaliplatin) for 3 months is the cost‐effective strategy and should be recommended in South Africa.

## Graphical abstract

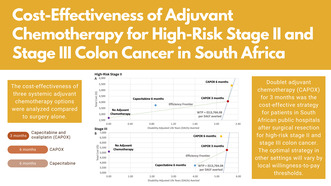

## 1 INTRODUCTION

The global incidence of cancer is expected to increase by 47% between 2020 and 2040, with even larger increases of 64%–95% predicted in low‐ and middle‐income countries (LMICs) experiencing rapid economic growth.^1^ This predicted rise is due to demographic changes, such as aging and population growth, as well as to increasing exposure to the main risk factors for cancer, several of which are associated with economic development.

Colon and rectal cancer incidence rates are a clear marker of economic development, with low incidence rates in many LMICs but sustained increases in countries undergoing rapid economic growth.^1^, ^2^ As countries in sub‐Saharan Africa (SSA) develop economically, colon and rectal cancer incidence on the sub‐continent is rising and placing financial burdens on resource‐constrained, publicly funded healthcare systems.^3^, ^4^, ^5^, ^6^ South Africa (ZA) is an upper‐middle income country and the most economically developed country in SSA. In ZA, colon and rectal cancers combined are the fourth most incident and sixth most lethal cancer.^7^ Between 2002 and 2014, the incidence of colon and rectal cancers increased consistently at a rate of 4.3% yearly for Black South African males, similar to trends noted in Eastern Europe and Latin America.^1^, ^5^

Post‐surgery adjuvant chemotherapy is standard of care for patients with high‐risk stage II and those with stage III colon cancer (CC) in high‐income countries (HICs). Resource‐stratified guidelines for the treatment of colon cancer patients in low resource settings also recommend adjuvant chemotherapy, but data is sparse on adherence to these guidelines among practitioners.^8^, ^9^, ^10^, ^11^ Additionally, a large cohort study of breast cancer patients treated across several public hospitals in ZA showed that 25% of eligible patients do not initiate adjuvant systemic therapy, despite availability at low or no cost.^12^ This data, despite being in a different tumor, suggest that adjuvant chemotherapy uptake is likely suboptimal in this setting.

In 2021, we evaluated the lifetime costs and effectiveness of treating patients with stage III CC with adjuvant chemotherapy, compared to surgery alone, in public hospitals in ZA.^13^ Here, we provide an updated model for patients with high‐risk stage II and stage III CC treated in ZA public hospitals, and present results from sub‐group analyses that identify the optimal treatment strategy according to patients' risk of CC recurrence. More than half of patients with CC in ZA present with stage II or III disease, so our updated model provides a more comprehensive understanding of the costs and benefits of adjuvant chemotherapy for patients with CC in ZA.^14^

As the number of cancer patients requiring treatment increases in LMICs like ZA, cost‐effectiveness research can help clinicians, public insurers, and the global oncology community understand the relative value and survival benefits of different treatment options. Importantly, policy makers can use cost‐effectiveness analyses to develop feasible and sustainable clinical treatment pathways. Such an approach has been associated with improved care and cost‐savings in HICs with public health systems.^15^, ^16^

## 2 METHODS

### 2.1 Model overview and inputs

The models utilized in this analysis were adapted from the model developed for patients with stage III CC in ZA, which is described in our previous publication.^13^ For this analysis, we updated the 2020 stage III CC model to reflect 2021 costs, expanded the model to include high‐risk stage II CC patients, and considered three adjuvant chemotherapy regimens: the combination of capecitabine and oxaliplatin (CAPOX) for 3 and 6 months; capecitabine for 6 months; and no adjuvant chemotherapy. Adjuvant chemotherapy regimens for CC that used 5‐fluorouracil (5‐FU) were found not to be cost‐effective in our original model and so were not considered in the updated model.^13^ The Markov models were developed using TreeAge Pro Healthcare 2021 software. Costs were considered from a societal perspective.

We used the National Comprehensive Cancer Network (NCCN) Guidelines to define high‐risk stage II CC as having any one of the following poor prognostic features: T4 disease, presence of lymphovascular or perineural invasion, presentation with obstruction or perforation, or having <10 lymph nodes examined during surgery.^17^

**FIGURE 1:**
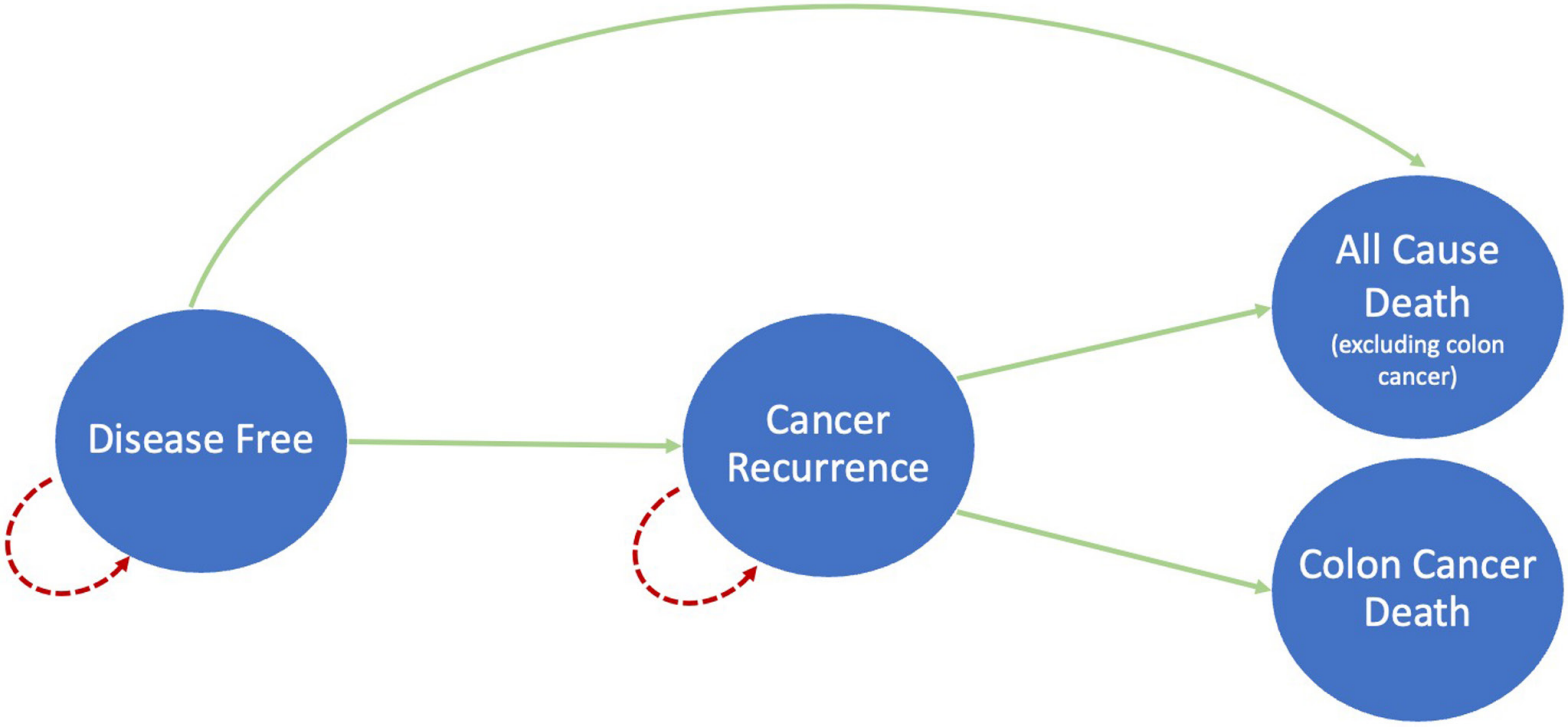
Markov model state transition diagram. Structure of the decision‐analytic Markov tree constructed and utilized to evaluate the cost‐effectiveness of adjuvant treatments for high‐risk stage II and stage III colon cancer patients in the South African public healthcare setting, showing the cohort entering the model in the disease‐free state post‐surgical resection, transitioning to various cancer recurrence states, and eventually ending in one of two death states: death from all other causes excluding colon cancer or death from colon cancer.

Our models simulated colon cancer recurrence and death following curative resection in a hypothetical cohort of 60‐year‐old ZA patients diagnosed with high‐risk stage II and stage III CC. Figure 1 and Tables S1–S4 of this manuscript and Table S2 of our prior publication^13^ provide detailed information on the model structure, inputs, and assumptions. Briefly, all patients started in the disease‐free state and could either remain disease‐free or transition to CC recurrence, death from CC, or death from all other causes excluding CC (Figure 1).^13^ We assumed that all CC recurrences were clinical distant metastases, one third of recurrences were liver‐only, and one third of patients with liver‐only CC recurrence underwent curative‐intent hepatectomy.^18^ Based on local practices, our models do not include the use of biologic agents in the metastatic setting and allow for a maximum of two lines of chemotherapy per patient (Figure S1A).^19^ The models ran for 300 months or until age 85 years based on the life expectancy in ZA.^20^ The model cycle length was 1 month and we applied a half‐cycle correction.

A summary of the key model inputs for the high‐risk stage II and stage III CC simulation models are described in Table 1. All transition probabilities were obtained from published literature. CC recurrence rates were estimated from disease‐free survival (DFS) and recurrence‐free curves from randomized controlled trials (RCTs), as previously described in our prior publication and as detailed in Table S1 and Figures S2 and S3.^13^ For the high‐risk stage II CC model, we estimated survival after CC recurrence using a published sub‐group analysis of the Multicenter International Study of Oxaliplatin/Fluorouracil/Leucovorin in the Adjuvant Treatment of Colon Cancer (MOSAIC) trial.^36^ Kaplan–Meier curves estimating survival after CC recurrence stratified by initial adjuvant treatment (doublet vs. single agent or no adjuvant chemotherapy) were traced using Engauge Digitizer Software and converted into monthly probabilities.^51^

**TABLE 1:** Key model and cost inputs in the South African high‐risk stage II and stage III colon cancer adjuvant chemotherapy simulation model.

Variable	Base case	Source
**Model parameters**		
Start age, years	60	21, 22, 23, 24, 25, 26
Annual discount rate	0.05	27, 28, 29, 30
Willingness‐to‐pay, I$ [ZAR]	13,764 [97,678]	31, 32, 33, 34
***CAPOX 3 months***		
*High‐risk stage II*		
Colon cancer recurrence probability (per month)	0.0002–0.0035	35
Colon cancer death probability (per month)	Tournigand et al. (2012, Figure 2D) (FOLFOX4)	36
*Stage III*		
Colon cancer recurrence probability (per month)	0.0014–0.0088	22, 37
Colon cancer death probability (per month)	Salem et al. (2020, Figure S1C) (1998–2003)	21
*Treatment costs, I$ [ZAR] (per treatment course)*		
Adjuvant treatment	1374 [9748]	19, 38, 39, 40, 41, 42, 43, 44, 45
First‐line metastatic treatment	8256 [58,586]	19, 38, 39, 40, 41, 42, 43, 44, 45, 46
***CAPOX 6 months***		
*High‐risk stage II*		
Colon cancer recurrence probability (per month)	0.0002–0.0034	35
Colon cancer death probability (per month)	Tournigand et al. (2012, Figure 2D) (FOLFOX4)	36
*Stage III*		
Colon cancer recurrence probability (per month)	0.0017–0.0101	22, 37
Colon cancer death probability (per month)	Salem et al. (2020, Figure S1C) (1998–2003)	21
*Treatment costs, I$ [ZAR] (per treatment course)*		
Adjuvant treatment	2747 [19,496]	19, 38, 39, 40, 41, 42, 43, 44, 45
First‐line metastatic treatment	8256 [58,586]	19, 38, 39, 40, 41, 42, 43, 44, 45, 46
***Capecitabine 6 months***		
*High‐risk stage II*		
Colon cancer recurrence probability (per month)	0.0011–0.0047	36
Colon cancer death probability (per month)	Tournigand et al. (2012, Figure 2D) (FL)	36
*Stage III*		
Colon cancer recurrence probability (per month)	0.0015–0.0141	26
Colon cancer death probability (per month)	Salem et al. (2020, Figure S1C) (1998–2003) and Tournigand et al. (2012, Figure 2D) (HR = 1.61)	21, 36
*Treatment costs, I$ [ZAR] (per treatment course)*		
Adjuvant treatment	1876 [13,314]	19, 38, 39, 40, 41, 42, 43, 44, 45
First‐line metastatic treatment	1869 [13,266]	19, 38, 39, 40, 41, 42, 43, 44, 45, 46
***No adjuvant chemotherapy***		
*High‐risk stage II*		
Colon cancer recurrence probability (per month)	0.0016–0.0052	11
Colon cancer death probability (per month)	Tournigand et al. (2012, Figure 2D) (FL)	36
*Stage III*		
Colon cancer recurrence probability (per month)	0.0021–0.0284	23
Colon cancer death probability (per month)	Salem et al. (2020, Figure S1C) (1998–2003) and Tournigand et al. (2012, Figure 2D) (HR = 1.61)	21, 36
*Treatment costs, I$ [ZAR] (per treatment course)*		
First‐line metastatic treatment	2804 [19,899]	19, 38, 39, 40, 41, 42, 43, 44, 45
Second‐line metastatic treatment	8256 [58,586]	19, 38, 39, 40, 41, 42, 43, 44, 45, 46
***All arms***		
**Probabilities**		
All cause death (excluding colon cancer)	2019 South Africa WHO life table (total population death)	20
**Direct costs, I$ [ZAR]**		
Hepatectomy (per procedure)^a^	8219 [58,329]	39, 40
*Surveillance*		
Bloodwork	6 [43]	40, 45
CT scan	318 [2260]	40, 45
Colonoscopy	315 [2233]	40, 45
*Grade 3/4 adverse events (per event)*		
Peripheral neuropathy		
During treatment	7 [51]	19, 38, 39, 40, 42
0–3 years post‐treatment (per year)^b^	29 [206]	19, 38, 39, 40, 42
Diarrhea	1105 [7485]	19, 38, 39, 40, 42
Febrile neutropenia	2455 [17,425]	19, 38, 39, 40, 42
Nausea and vomiting	1110 [7877]	19, 38, 39, 40, 42
Mucositis	1117 [7924]	19, 38, 39, 40, 42
Hand‐foot syndrome	1117 [7928]	19, 38, 39, 40, 42
**Indirect costs, I$ [ZAR] (per day)** ^c^		
Transportation	6 [41]	12
Lost wages	20 [138]	47, 48, 49
**Annual disability weights**		
*Health states*		
Disease free	0	‐
Colon cancer recurrence (metastatic)	0.451	50
All cause death	NA	‐
Colon cancer death	1	‐
*Adverse events*		
Peripheral neuropathy	0.133	50
Diarrhea	0.247	^50^
Febrile neutropenia	0.133	^50^
Nausea and vomiting	0.114	50
Mucositis	0.051	50
Hand‐foot syndrome	0.133	50

Abbreviations: CAPOX, capecitabine and oxaliplatin; CT, computed tomography; FL, fluorouracil and leucovorin; FOLFOX4, infusional fluorouracil, leucovorin, and oxaliplatin; HR, hazard ratio; I$, international dollars (2021); NA, not applicable; WHO, World Health Organization; ZAR, South African rand (2021).

^a^ Hepatectomy cost encompasses the costs of the surgical procedure; hospitalization, including intensive care unit (ICU) admission, for post‐surgery recovery; and outpatient follow‐up.

^b^ Grade 2/3 residual neuropathy.

^c^ Indirect costs for time spent for each surveillance CT scan (applied per patient); colonoscopy (applied per patient and one caregiver per patient); and adjuvant, first‐line metastatic, and second‐line metastatic treatment (applied per patient and one caregiver per patient).

For the stage III CC model, we estimated survival after CC recurrence using data from a published analysis of the adjuvant colon cancer end points (ACCENT) database.^13^, ^21^ We used data from patients with stage III CC enrolled between 1998 and 2003 to more closely approximate current treatment patterns in ZA. Assuming that initial adjuvant treatment had a similar effect on survival after CC recurrence in high‐risk stage II and stage III CC, we used the hazard ratio (HR) of 1.61 from the MOSAIC trial subgroup analysis for high‐risk stage II CC to adjust survival after recurrence for patients with stage III CC who received no chemotherapy or capecitabine in the adjuvant setting.^36^ Internal validation of the model was done by comparing model DFS outputs to 5‐year DFS data from the respective RCTs used to calculate CC recurrence rates. All 5‐year DFS model outputs were within 1.5% of the 5‐year DFS outputs from the RCTs used for model construction.

All costs were calculated utilizing the South African Medicine Price Registry's Database of Medicine Prices and ZA Department of Health's 2021 Uniform Patient Fee Schedule (Tables S2–S4).^38^, ^39^ All costs were converted from 2021 South African rand (ZAR) to international dollars (I$) using the 2021 purchasing power parity (PPP) (conversion factor: ZAR/7.097 PPP = I$).^31^

### 2.2 External model validation

To externally validate our model, model predictions for OS were compared to data from the colorectal cancer in South Africa (CRCSA) study, prospective cohort study conducted in five hospitals in Johannesburg, ZA from 2016 to 2019, which was not used for model development.^52^ There were 55 stage II CC and 79 stage III CC patients enrolled in the CRCSA study, with a median follow‐up time of 54 and 41 months, respectively. Model OS predictions fit within the 95% CI of the CRCSA OS estimates at 12, 34, and 36 months (Table S5).

### 2.3 Outcomes

Our primary outcomes were death from CC and disability due to CC recurrence and/or treatment‐related adverse events (TRAEs). We report these primary results as the incremental cost‐effectiveness ratio (ICER) in I$ per disability‐adjusted life‐year (DALY) averted. For each treatment strategy, DALYs were calculated as the sum of the years of life lost (YLL) and years lived with disability (YLD) due to colon cancer.^53^ Model recommendations reference a base case willingness‐to‐pay (WTP) threshold equal to the 2021 Gross Domestic Product (GDP) per capita of ZA (I$13,764).^32^, ^33^, ^34^ Overall survival (OS) is reported as undiscounted and unadjusted life years. All other cost‐effectiveness analyses are discounted using a 5% annual rate and adjusted for disability, without age weighting.^27^, ^28^, ^54^

A secondary model output for each treatment strategy was the net monetary benefit (NMB), calculated using the formula:

NMB=WTP*Effectiveness−Cost

### 2.4 Sensitivity, subgroup, and scenario analyses

In deterministic sensitivity analyses, key model variables were varied one at a time according to ranges reported in the literature and common practices in economic evaluations.^55^, ^56^, ^57^ In a one‐way deterministic sensitivity analysis, we varied the start age in our models from 40 to 80 years to assess the impact of early onset CC on the cost‐effective treatment strategy. In a probabilistic sensitivity analysis (PSA) with 100,000 random iterations, all key variables were varied simultaneously, as well as the WTP threshold from one‐half (I$6882) to three times (I$41,292) the ZA GDP per capita.^33^, ^54^, ^58^, ^59^, ^60^ Table 2 summarizes the variables, distributions, and ranges used in the deterministic tornado and probabilistic sensitivity analyses.

**TABLE 2:** Deterministic tornado and probabilistic sensitivity analysis parameters for the high‐risk stage II and stage III colon cancer models.

Variable	Deterministic sensitivity analysis range	Probabilistic sensitivity analysis		Source
		Distribution	Range (SD)	
Annual discount rate	0.0–0.10	N/A	N/A	27, 28, 29, 30
Willingness‐to‐pay	−50% to +200%	N/A	N/A	31, 32, 34
*Probabilities*				
Colon cancer death	−25% to +25%	β	10%	21, 36
All cause death (excluding colon cancer)	2019 South Africa WHO life table female to male death	*β*	10%	20
Colon cancer recurrence	−25% to +25%	*β*	10%	11, 22, 23, 26, 35, 36, 37
Adverse events	−25% to +25%	*β*	10%	26, 46, 61
*Costs*				
Adjuvant treatment	−50% to +100%	*γ*	25%	11, 12, 19, 22, 26, 35, 36, 37, 38, 41, 42, 43, 44, 47, 48, 49
First‐line metastatic treatment	−50% to +100%	*γ*	25%	11, 12, 19, 22, 26, 35, 36, 37, 38, 41, 42, 43, 44, 47, 48, 49
Second‐line metastatic treatment	N/A	*γ*	25%	11, 12, 19, 22, 26, 35, 36, 37, 38, 41, 42, 43, 44, 47, 48, 49
*Annual disability weights*				
Colon cancer recurrence	0.307–0.600	PERT	0.307–0.600	50
Peripheral neuropathy	0.089–0.187	PERT	0.089–0.187	50
Diarrhea	0.164–0.348	PERT	0.164–0.348	50
Febrile neutropenia	0.088–0.190	PERT	0.088–0.190	50
Nausea and vomiting	0.078–0.159	PERT	0.078–0.159	50
Mucositis	0.032–0.074	PERT	0.032–0.074	50
Hand‐foot syndrome	0.078–0.159	PERT	0.078–0.159	50

Abbreviations: *β*, beta distribution; FOLFIRI, fluorouracil, leucovorin, and irinotecan; *γ*, gamma distribution; SD, standard deviation; WHO, World Health Organization; XELIRI, capecitabine and irinotecan.

We performed subgroup analyses for both the high‐risk stage II and stage III CC models. For the high‐risk stage II model, we analyzed patients by tumor stage (T4 vs. T3 stage) and number of lymph nodes evaluated (< 10 vs. ≥10 lymph nodes). Subgroup‐specific recurrence‐free survival values obtained from the Personalized Adjuvant TreaTment in EaRly stage coloN cancer (PATTERN) model were used to adjust the probability of recurrence inputs used in the base case high‐risk stage II model (Table S6a).^62^ For the stage III model, we analyzed patients by colon cancer recurrence risk, as defined in the International duration evaluation of adjuvant therapy (IDEA) collaboration. Patients with T3N1 stage III CC were considered low risk while patients with stage III CC and T4 or N2 disease were considered high‐risk for CC recurrence. Risk‐specific recurrence probabilities were derived from the IDEA trials (Table S6b).^22^, ^63^

Lastly, we performed a scenario analysis for the high‐risk stage II and stage III CC models to determine if model recommendations change when all instances of treatment with 5‐FU are replaced with capecitabine. In the base case scenario, we allowed for leucovorin, 5‐FU, and irinotecan (FOLFIRI) to be used in the first‐ and second‐line metastatic setting; in the capecitabine‐only scenario analysis only capecitabine and irinotecan (XELIRI) was allowed (Figure S1A). The capecitabine‐alone scenario allowed us to model an all‐capecitabine‐based systemic treatment scenario, which may be preferable in the ZA public healthcare system due to the high financial cost of 5‐FU‐based treatments.

## 3 RESULTS

### 3.1 Base case cost‐effectiveness analysis

Model results for adjuvant treatment of patients with high‐risk stage II and stage III CC, including the total cost, unadjusted OS, and DALYs averted associated with each treatment, are shown in Table 3. Model DFS outputs closely approximated the values reported in RCTs (Figures S2 and S3). For a cohort of 60‐year‐old patients with high‐risk stage II CC in the ZA public healthcare sector, the unadjusted OS without adjuvant chemotherapy was 13.69 years. The cost‐effective treatment strategy was CAPOX for 3 months (CAPOX 3MO), with an unadjusted OS of 15.33 years, a lifetime cost of I$4083, and 2.21 DALYs averted per patient compared to no adjuvant chemotherapy. For a cohort of 60‐year‐old patients with stage III CC in the same setting, the unadjusted OS without adjuvant chemotherapy was 9 years. The cost‐effective strategy was also CAPOX 3MO, with an unadjusted OS of 12.99 years, a lifetime cost of I$5279 and 5.46 DALYs averted per patient compared to no adjuvant chemotherapy. In both cases, all strategies had a positive NMB except for no adjuvant chemotherapy, reflecting that all strategies are cost‐effective compared to surgery alone. CAPOX 3MO had the highest NMB of I$26,320 for patients with high‐risk stage II and I$69,921 for stage III CC.

**TABLE 3:** Cost‐effectiveness results from the South African high‐risk stage II and stage III colon cancer adjuvant chemotherapy simulation models, with no adjuvant chemotherapy as comparator and a willingness‐to‐pay (WTP) threshold equal to South Africa's 2021 GDP per capita (base case scenario).

Strategy	Total cost, I$ [ZAR]	Overall survival, years	DALYs averted	ICER, I$/DALY averted	NMB, I$ [ZAR]
*High‐risk stage II*					
No adjuvant chemotherapy	2693 [19,111]	13.69	0.00	‐	−2692 [−19,107]
Capecitabine 6 months	3748 [26,598]	14.34	0.87	Extended dominated	8221 [58,338]
CAPOX 3 months	4083 [28,974]	15.33	2.21	250	26,320 [186,789]
CAPOX 6 months	5398 [38,312]	15.39	2.28	18,421	25,987 [184,429]
*Stage III*					
Capecitabine 6 months	3737 [26,522]	11.87	3.98	‐	51,096 [362,628]
No adjuvant chemotherapy	5117 [36,316]	9.00	0.00	Dominated	−5144 [−36,509]
CAPOX 3 months	5279 [37,463]	12.99	5.46	1042	69,921 [496,233]
CAPOX 6 months	6647 [47,172]	12.79	5.19	Dominated	64,857 [460,289]

*Note*: Overall survival is presented as life years, undiscounted and unadjusted for disability. All other results have a global annual discounting of 5% (*r* = 0.05) applied to all costs and effectiveness calculations and are adjusted using annual disability weights.

Abbreviations: CAPOX, capecitabine and oxaliplatin; DALY, disability‐adjusted life‐year; GDP, gross domestic product; ICER, incremental cost‐effectiveness ratio; I$, international dollars (2021); NMB, net monetary benefit; ZAR, South African rand (2021).

**FIGURE 2:**
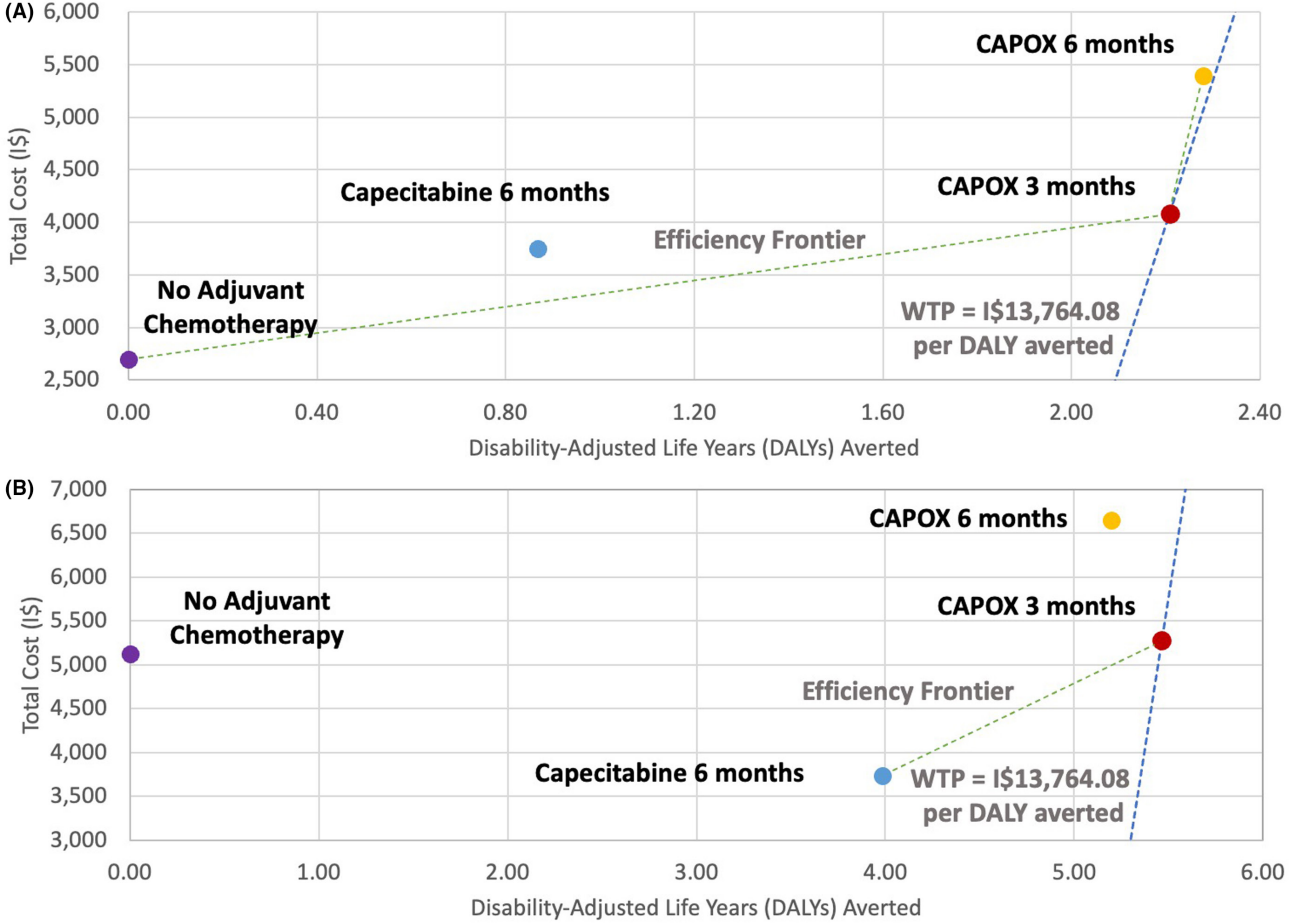
Base case cost‐effectiveness analysis of three adjuvant therapy regimens compared to receiving no adjuvant chemotherapy for (A) high‐risk stage II and (B) stage III colon cancer. The figure shows that only two treatment strategies: CAPOX for 3 months and CAPOX for 6 months for high‐risk stage II; and CAPOX for 3 months and Capecitabine for 6 months for stage III are undominated and lie on the efficiency frontier (green dashed line). For both high‐risk stage II and stage III colon cancer, only CAPOX for 3 months is cost‐effective. For high‐risk stage II, CAPOX for 6 months is not cost‐effective as the ICER exceeds the WTP threshold (blue dashed line). CAPOX, capecitabine and oxaliplatin; DALY, disability‐adjusted life‐year; I$, international dollars (2021); ICER, incremental cost‐effectiveness ratio; WTP, willingness‐to‐pay.

For the high‐risk stage II CC model, CAPOX for 6 months (CAPOX 6MO) was on the efficiency frontier, with an incrementally higher effectiveness than the optimal strategy (0.07 additional DALYs averted) but a higher lifetime cost (I$5398). The ICER of I$18,421/DALY averted for CAPOX 6MO was above the WTP threshold of I$13,764 and the NMB (I$25,987) was lower than that for CAPOX 3MO. All other adjuvant treatment strategies were dominated (Figure 2A). For the stage III CC model, CAPOX 3MO was the only undominated strategy (Figure 2B).

Our models also predicted that oxaliplatin‐based adjuvant chemotherapy is more effective than single‐agent capecitabine, regardless of stage (OS 15.33–15.39 years vs. 13.69 years for high‐risk stage II and OS 12.79–12.99 years vs. 11.87 years for stage III CC).

### 3.2 Sensitivity analyses

**FIGURE 3:**
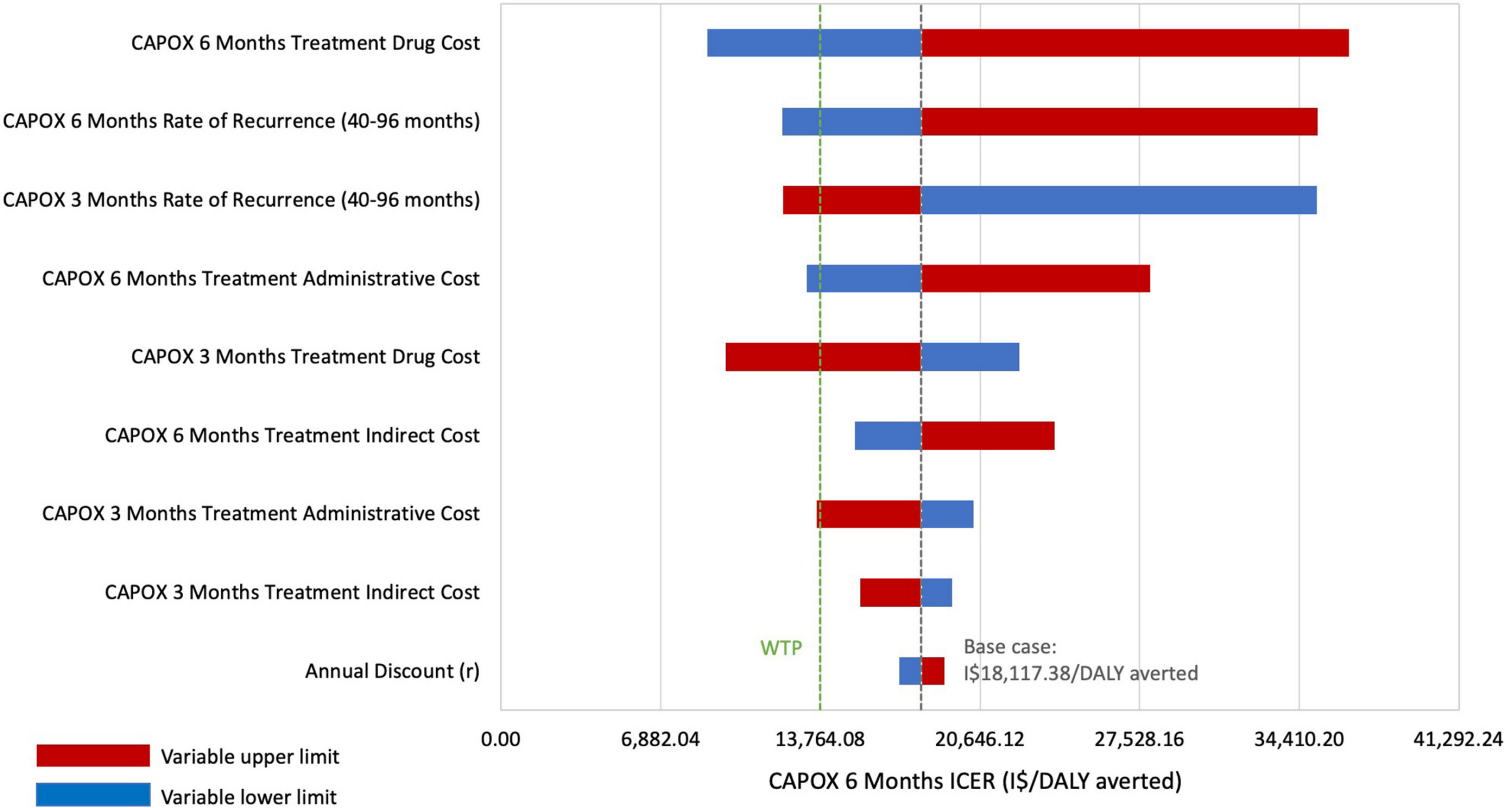
One‐way deterministic sensitivity analysis ICER tornado diagram for high‐risk stage II colon cancer. This tornado diagram illustrates the results of a one‐way sensitivity analysis of key model inputs. The plot shows the impact of varying these parameters on the ICER of CAPOX for 6 months, the only other strategy on the efficiency frontier for high‐risk stage II colon cancer, in comparison to CAPOX for 3 months, the optimal strategy. CAPOX, capecitabine and oxaliplatin; DALY, disability‐adjusted life‐year; ICER, incremental cost‐effectiveness ratio; I$, international dollars (2021); WTP, willingness‐to‐pay.

Deterministic sensitivity analyses indicated that out models' recommendations remained unchanged when accounting for uncertainty in key model parameters. We present these results for the two strategies on the efficiency frontier in the high‐risk stage II model: CAPOX 3MO and CAPOX 6MO (Figure 3). The drug cost of adjuvant treatment with CAPOX 6MO had the greatest effect on model outcomes. If the drug cost associated with adjuvant treatment for CAPOX 6MO decreased below 76% or the probability of CC recurrence decreased below 83% of the base case value, the ICER for CAPOX 6MO may fall below the WTP threshold and become cost‐effective. For the stage III model, Capecitabine for 6 months was the reference strategy, with the lowest lifetime cost of I$3737 and 3.98 DALYs averted, and CAPOX 6MO was dominated; thus neither strategy ever becomes cost‐effective. For these reasons, we do not report a tornado diagram for the stage III model.

We conducted an additional one‐way sensitivity analysis to examine the impact of the average age of the patient cohort at the start of each model. We found that even when ranging the start age between 40 and 80 years, the ICER for CAPOX 6MO compared to CAPOX 3MO for high‐risk stage II CC remains above the WTP threshold. Even at a start age of 40 years, to account for a higher incidence of early onset CC in Black South Africans,^5^ the ICER for CAPOX 6MO is above I$18,100/DALY averted and is therefore never cost‐effective. For stage III, the ICER for CAPOX 3MO compared to capecitabine for 6 months remains well below the WTP threshold, ranging from approximately I$1080/DALY averted at age 40 years to I$800/DALY averted at age 80 years, so CAPOX 3MO is always cost‐effective.

We performed the PSA at a WTP threshold equal to the 2021 GDP per capita and accounted for uncertainty in all key model variables. CAPOX 3MO remained optimal for 74% of iterations for the high‐risk stage II and 79% of iterations for the stage III model. Figure S4 depicts the distribution of results for each of the 100,000 random iterations, and Figure S5 displays the cost‐effectiveness acceptability curve for a WTP range equal to one half to three times the base case WTP threshold.

### 3.3 Subgroup analyses

The results of the analysis stratifying patients with high‐risk stage II colon cancer by T staging are presented in Table S7. CAPOX 6MO was the optimal strategy for patients with high‐risk stage II and T4 disease with 3.50 DALYs averted, while CAPOX 3MO remained the optimal strategy for patients with T3 disease with 2.04 DALYs averted. In a second subgroup analysis we show that CAPOX 3MO remains the optimal strategy for patients with high‐risk stage II CC, regardless of number of lymph nodes evaluated during surgery (Table S8).

Results for the analysis of patients with stage III CC stratified by risk of CC recurrence are presented in Table S9. CAPOX 3MO remained the optimal strategy in the low‐risk stage III CC subgroup, defined as having T3 N1 disease (7.37 DALYs averted), while CAPOX 6MO became optimal in the high‐risk stage III CC subgroup, defined as having T4 or N2 disease (7.36 DALYs averted).

### 3.4 Scenario analysis

CAPOX 3MO remained the optimal strategy for both patients with high‐risk stage II CC and patients with stage III CC when XELIRI was used in the metastatic setting for all patients who received either CAPOX in the adjuvant setting or no adjuvant treatment with 2.21 DALYs averted for high‐risk stage II and 5.46 DALYs averted for stage III CC (see Table S10).

## 4 DISCUSSION

The global public health burden of CC is substantial; it is estimated that 70% of colon and rectal cancer‐related deaths worldwide occur in LMICs,^52^ where late stage at presentation is common. In HICs, adjuvant chemotherapy after surgical resection of locally advanced (high‐risk stage II and stage III) CC is standard of care^17^, ^64^; these guidelines are informed by RCT data showing that adjuvant chemotherapy is associated with a 20%–30% relative reduction in CC recurrence in high‐risk stage II and stage III CC.^62^, ^65^, ^66^, ^67^ However, most patients with locally advanced CC are diagnosed and treated in LMICs, where several complex factors, including cost, result in variable access to and receipt of adjuvant chemotherapy.

ZA is an upper‐middle income country with a robust public healthcare system. Approximately 85% of the population receives heavily subsidized or free healthcare, including cancer care, through a network of public hospitals.^19^ Given a steady increase in CC incidence in ZA since 2002, the patient population eligible for adjuvant chemotherapy after surgical resection of CC is also steadily increasing.^5^ This is likely to place a substantial burden on the public healthcare system in ZA and in other LMICs experiencing similar rises in CC incidence. In this study, we adapted a previously developed decision‐analytic Markov model for stage III CC in ZA^13^ and used it to determine the cost‐effective adjuvant treatment strategy for patients with high‐risk stage II and patients with stage III CC after surgical resection. Few studies have compared the value of different adjuvant treatment strategies following surgical resection of CC in resource‐limited health systems, and to our knowledge, there are no other published studies that offer a comprehensive cost‐effectiveness analysis for adjuvant CC treatment in ZA.

Our main results can be considered in two parts. We found that for high‐risk stage II and stage III CC patients in ZA public hospitals who have undergone surgical resection: (1) doublet adjuvant chemotherapy (CAPOX) is cost‐effective, compared to no adjuvant chemotherapy (observation) and single‐agent adjuvant chemotherapy (capecitabine); and (2) doublet chemotherapy for 3 months (CAPOX 3MO) is cost‐effective compared to no adjuvant chemotherapy, doublet chemotherapy for 6 months (CAPOX 6MO), and single‐agent chemotherapy for 6 months. These results are largely consistent with treatment guidelines in the United States (US) and Europe, with previously published cost‐effectiveness analyses from HICs, and with our prior analysis limited to stage III CC patients in ZA.^13^

With regard to doublet versus observation or single agent adjuvant chemotherapy in stage III CC, our results are consistent with results from RCTs^23^, ^65^ and several prior cost‐effectiveness analyses. For example, Shiroiwa et al. and Aballéa et al. both found that adjuvant fluorouracil, leucovorin, and oxaliplatin (FOLFOX) is cost‐effective compared to 5‐FU/leucovorin for patients with stage III CC in Japan and the US, with an ICER of US$17,000 and US$22,800 per quality‐adjusted life‐year (QALY) respectively.^68^, ^69^

The effectiveness and cost‐effectiveness of adjuvant chemotherapy (single agent or doublet) versus observation for high‐risk stage II CC has not been consistently demonstrated in the literature, and there is great variation in treatment patterns worldwide.^70^, ^71^ The QUick and Simple And Reliable (QUASAR) trial randomly assigned patients with resected stage II colon or rectal cancer (without stratification by risk of recurrence) to observation versus adjuvant chemotherapy with 5‐FU; in this trial, adjuvant chemotherapy was associated with a 22% decrease in the risk of recurrence (*p* < 0.001), although the study has been criticized for not presenting data for CC patients separately.^67^ A subgroup analysis of the MOSAIC trial showed that patients with high‐risk stage II CC randomized to FOLFOX versus 5‐FU had improved 5‐year relapse free survival (86.8% [95% CI, 82.2–90.3] vs. 78.8% [95% CI, 73.4–83.2]), but no difference in DFS or OS.^36^ A decision analysis study comparing the cost‐effectiveness of adjuvant chemotherapy with 5‐FU versus observation and FOLFOX versus 5‐FU in patients who have undergone resection for stage II CC in the US using data from MOSAIC and QUASAR found that doublet adjuvant chemotherapy is cost‐effective with an ICER of US$54,359/QALY for the base‐case scenario of 60‐year‐old patients. The ICER for FOLOX decreased to US$39,225/QALY in a sensitivity analysis for 50‐year‐old patients.^72^ Our model, using data from MOSAIC, similarly found doublet adjuvant chemotherapy to be cost‐effective in our base‐case scenario of 60‐year‐old patients with high‐risk stage II CC in ZA. Our results remained unchanged when the model start age was decreased to 40 years in a one‐way sensitivity analysis.

With regard to duration of therapy, our findings are consistent with those from the short course oncology therapy trial, which found that 3 months of the doublet adjuvant chemotherapy regimen was cost‐effective compared to 6 months in high‐risk stage II or stage III colon and rectal cancer in the UK, Denmark, Spain, Sweden, Australia, and New Zealand.^73^ Jongeneel et al. also found that, compared to 6 months of CAPOX or 3 or 6 months of FOLFOX, 3 months of CAPOX was the cost‐effective adjuvant treatment for patients with high‐risk stage II CC and recommended that this become the standard of care in the Netherlands.^74^ Although no national treatment pathway for adjuvant treatment of colon cancer has been adopted in the ZA public healthcare system, Herbst et al. used published clinical trial data, discussions with Medical Oncologists in a Public Academic Hospital, the Department of Health Master Procurement Catalog (equivalent to the local Essential Medicines List) for approved chemotherapy medications available in the ZA public healthcare sector, and real‐world data from a public hospital to develop and publish a proposed treatment algorithm for non‐metastatic CC in the ZA public healthcare system. This treatment algorithm recommends 6 months of doublet chemotherapy for high‐risk stage 3 CC, capecitabine monotherapy for low‐risk stage 3 CC, and makes no recommendations for adjuvant therapy in stage 2 CC. We believe our model provides evidence that can be used to update this treatment algorithm, and to encourage its use across public hospitals in ZA so that the greatest number of patients can benefit using the currently available resources.

The models used in this study were adapted from our model published in 2021, which simulated disease progression in patients with stage III CC treated in ZA public hospitals. For this study, we expanded our model to include patients with high‐risk stage II CC and eliminated adjuvant chemotherapy strategies that used 5‐FU (single‐agent 5‐FU and FOLFOX for 3 or 6 months). By including patients with high‐risk stage II CC, we now provide a comprehensive analysis of adjuvant CC treatment in the ZA public healthcare setting, which has not been previously attempted. The decision to eliminate 5‐FU‐based adjuvant strategies was informed by our 2021 results, which demonstrate that 5‐FU and FOLFOX are never cost‐effective in the ZA public healthcare system, largely due to the high fixed costs associated with placing a port‐a‐cath and using a continuous infusion pump. Further, we updated the survival after CC recurrence data used in this model to account for improved survival in patients who have not received adjuvant chemotherapy or received single agent therapy compared to those who experienced a recurrence after doublet adjuvant chemotherapy.^36^ Despite this change, doublet chemotherapy upfront remained the more effective and cost‐effective strategy for patients with high‐risk stage II and stage III CC in the ZA public healthcare setting. Importantly, we used clinical data from a prospective cohort study of colorectal cancer patients treated in Johannesburg, ZA to externally validate model outputs. Although the dataset used for external validation was small model OS predictions all fell within the 95% CI of survival estimates of this real‐world data, lending further credibility to model results and recommendations (Table S5).

We performed several subgroup analyses to determine whether there are groups of patients for whom treatment de‐escalation (single agent capecitabine) or escalation (6 months of CAPOX) is the optimal strategy. We found that for patients with high‐risk stage II CC with T4 tumors, CAPOX 6MO is the optimal strategy due to a higher risk of recurrence in this group compared to patients with T3 tumors.^62^ In patients with high‐risk stage II CC with T3 tumors, CAPOX 3MO remained the optimal strategy; and despite a lower risk of recurrence in this group, the adjuvant treatment strategy of single agent capecitabine was dominated. The optimal adjuvant treatment strategy did not change based on the number of harvested/examined lymph nodes during surgical resection for high‐risk stage II CC (<10 vs. ≥10 lymph nodes). For patients with stage III CC, we performed a subgroup analysis based on CC recurrence risk groups as defined by the IDEA trial.^22^ For patients with T3N1 disease, CAPOX 3MO remained the optimal strategy while for patients with T4 or N2 disease who are at higher risk for CC recurrence, CAPOX 6MO became optimal. These results are consistent with our previously published model and with treatment guidelines in the US and Europe.^17^, ^22^, ^37^, ^64^

There are limitations to our study. These include the model's assumption that patient outcomes from RCTs performed in HICs with majority White populations are representative of those in the ZA public healthcare setting serving a majority Black population and the absence of long‐term follow‐up data for certain treatment arms. Our sensitivity tests indicate that our results are robust to variations in these assumptions, and more detailed descriptions of our approach to addressing these limitations are included in our prior publication.^13^

Health disparities specific to ZA further complicate interpretation of our results. Black ZAs present with CC at younger ages than the average age in RCT populations from HICs and the age‐standardized incidence and mortality rates rose faster for Black men than for any other demographic group in ZA.^5^ Our model does not consider race specifically, which is a proxy measure of both access to healthcare and outcome disparities by socioeconomic status, but our overall sensitivity analysis suggests that the cost‐effective treatment strategy is the same across patient groups. The sensitivity analysis does consider disease recurrence rates, which we would expect to be higher among patients with less access to care. CAPOX 3MO remains the cost‐effective treatment strategy even when we vary the recurrence rate by ±25%, as shown in our deterministic and PSA results. Additionally, our one‐way sensitivity analysis shows that even for patients who develop CC at age 40 years, the cost‐effective strategy remains CAPOX 3MO. Our approach to these limitations demonstrates that cost‐effectiveness modeling in LMICs can be done using clinical trial data from HICs, but fewer assumptions would be necessary if clinical trial data were available from LMICs.

In conclusion, this is the first comprehensive model‐based study that evaluates the cost‐effectiveness of adjuvant chemotherapy for locally advanced CC in ZA, and few similar studies exist in other LMICs. Our results show that CAPOX for 3 months is the cost‐effective adjuvant chemotherapy treatment strategy for patients in ZA public hospitals with high‐risk stage II and stage III CC. This study demonstrates that decision modeling can help health systems in low resource settings select cost‐effective treatments for their growing population of cancer patients.

## AUTHOR CONTRIBUTIONS

**Sarah Xinhui Tan:** Formal analysis (lead); investigation (lead); methodology (lead); project administration (lead); writing – original draft (lead); writing – review and editing (lead). **Yoanna Pumpalova:** Conceptualization (lead); formal analysis (supporting); funding acquisition (equal); supervision (lead); writing – original draft (supporting); writing – review and editing (equal). **Alexandra Rogers:** Formal analysis (supporting); investigation (supporting); methodology (supporting). **Kishan Bhatt:** Writing – original draft (equal); writing – review and editing (equal). **Candice‐lee Herbst:** Data curation (equal); investigation (supporting); methodology (supporting). **Paul Ruff:** Conceptualization (equal); writing – review and editing (supporting). **Alfred Neugut:** Conceptualization (equal); writing – review and editing (supporting). **Chin Hur:** Conceptualization (lead); supervision (equal); validation (equal); writing – review and editing (supporting).

## FUNDING INFORMATION

This study received support from the Herbert Irving Comprehensive Cancer Center Support Grant awarded by the National Cancer Institute (NCI P30 CA013696). External validation of model outputs was performed using real‐world data from the Colorectal Cancer in South Africa (CRCSA) study, a prospective study funded by the Medical Research Council of South Africa, through the Wits/SAMRC Common Epithelial Cancer Research Centre (CECRC) Grant (Paul Ruff, principal investigator). Permission to use this dataset was obtained from the study principal investigator (Dr. Paul Ruff). *Role of the funding source*: The funders described had no role in the study design; data collection, analysis, or interpretation; or manuscript writing. All authors listed had full access to the data in the study and take final responsibility for the decision to submit this manuscript for publication.

## CONFLICT OF INTEREST STATEMENT

Dr. Neugut has received consulting fees from Otsuka, United Biosource Corp, Hospira, Eisai, and GlaxoSmithKline, and is on the medical advisory board of EHE Intl. He has also received research funding from Otsuka. Dr. Ruff has received honoraria from Sanofi, Amgen, and Roche and research funding from Amgen, Sanofi, Merck and Novartis. Dr. Hur has received consulting fees from Exact Sciences. Dr Pumpalova owns stock in Pfizer. All other authors have no conflicts of interest to declare.

## ETHICS STATEMENT

Ethical approval for retrieving the cost data associated with adjuvant colon cancer treatment was provided through the Human Research Ethics Committee at the University of Witswatersrand (M1409809).

## Supporting information


Data S1:

## Data Availability

The data generated during the current study are available from the corresponding author by request.
