# Supplementary material for: Cost‐effectiveness of adjuvant chemotherapy for high‐risk stage II and stage III colon cancer in South Africa
Source: Cancer Med. 2023 Jun 15;12(14):15515–29. doi: 10.1002/cam4.6199 (PMC10417185; doi:10.1002/cam4.6199)
Supplement: Supplementary file 1 — Data S1: [file CAM4-12-15515-s001.docx]

**Supplementary Material**

**Supplementary Table 1. Summary of results from clinical trials used to inform rates of disease progression and mortality in the base case for high-risk stage II and stage III colon cancer.**

|  | | | | |
| --- | --- | --- | --- | --- |
| Strategy | No Adjuvant Chemotherapy | Capecitabine 6 months | CAPOX 3 months | CAPOX 6 months |
| *Base Case High-Risk Stage II* | | | | |
| Trial | Kumar *et al* 2015 | MOSAIC 2012 | IDEA 2021 | IDEA 2021 |
| Number of participants | 913 | 1,123 | 1,020 | 999 |
| Median age, years | 72 | 61 | 64 | 64 |
| 5-year rate of DFS, % (95% CI) | 74.5*^‡^ | 78.5*^‡^ | 81.7 (79.2-84.3) | 82.0 (79.3-84.7) |
| Source | ^1^ | ^2^ | ^3^ | ^3^ |
| *Base Case Stage III* | | | | |
| Trial | Moertel *et al* 1995 | X-ACT 2005 | IDEA 2020 | IDEA 2020 |
| Number of participants | 315 | 1,004 | 2,549 | 2,515 |
| Median age, years | 60 | 62 | 65 | 65 |
| 5-year rate of DFS, % (95% CI) | 45.1*^‡^ | 62.4^‡^ | 70.0 (68.1-71.9) | 69.3 (67.4-71.3) |
| Source | ^4^ | ^5^ | ^6,7^ | ^6,7^ |

CAPOX=capecitabine and oxaliplatin. DFS=disease-free survival. CI=confidence interval. MOSAIC=Multicenter International Study of Oxaliplatin/Fluorouracil/Leucovorin in the Adjuvant Treatment of Colon Cancer. IDEA=International Duration Evaluation of Adjuvant Therapy. X-ACT=Xeloda in Adjuvant Colon Cancer Therapy.

*Reported as percent of patients free from recurrence.

^‡^5-year rate digitized from published Kaplan Meier curves; confidence intervals not reported.

**Supplementary Table 2. Direct costs associated with chemotherapy treatment regimens.**

| Chemotherapy component cost,  I$ [ZAR]^8^ | CAPOX 3 months | CAPOX 6 months | Capecitabine 6 months | XELIRI 4 months | FOLFIRI 4 months |
| --- | --- | --- | --- | --- | --- |
| Drug^9^ | 182.62 [1,295.99] | 182.62 [1,295.99] | 103.14 [731.95] | 243.16 [1,725.58] | 381.70 [2,708.75] |
| Personnel^¥10^ | 97.76 [693.73] | 97.76 [693.73] | 22.33 [161.04] | 97.76 [693.73] | 97.76 [693.73] |
| Anti-emetics^9,11^ | 4.46 [31.62] | 4.46 [31.62] | 1.92 [13.64] | 5.02 [35.66] | 5.02 [35.66] |
| Administration^‡10,12,13^ | 1.82 [12.92] | 1.82 [12.92] | 0.00 [0.00] | 1.82 [12.92] | 1,207.60 [8,569.81] |
| Bloodwork^14,15^ | 6.06 [43.00] | 6.06 [43.00] | 6.06 [43.00] | 6.06 [43.00] | 6.06 [43.00] |
| Treatment cost (per cycle) | 292.71 [2,270.05] | 292.71 [2,270.05] | 133.45 [947.04] | 353.82 [2,510.88] | 1,698.13 [12,050.94] |
| Number of days per cycle | 21 | 21 | 21 | 21 | 14 |
| Number of cycles^5,16,17^ | 4 | 8 | 8 | 6 | 8 |
| Total cost (per treatment course) | 1,170.85 [9,080.19] | 2,471.01 [16,618.07] | 1,067.60 [7,576.30] | 2,122.89 [15,065.28] | 13,585.08 [96,407.49] |

I$= international dollars (2021). ZAR=South African rand (2021). CAPOX=capecitabine and oxaliplatin. XELIRI=capecitabine and irinotecan. FOLFIRI=fluorouracil, leucovorin, and irinotecan.

^¥^Personnel costs include the costs for the physician, clerk, nurse, and pharmacist

^‡^Administration costs include costs of port-a-cath placement and continuous infusion pump for FOLFIRI and costs associated with peripheral IV access for all other regimens except Capecitabine

**Supplementary Table 3. Direct costs associated with grade 3/4 treatment-related adverse events.**^8,10,12,14^

| Adverse event | Cost component | Cost per dose, I$ [ZAR] | Doses per day | Duration of treatment, days | Total cost,  I$ [ZAR] |
| --- | --- | --- | --- | --- | --- |
| Peripheral neuropathy | | | | | |
| During treatment | Amitriptyline (25 mg) | 0.08 [0.57] | 1 | 90 | 7.17 [50.85] |
|  | Total (per treatment course) | | | | 7.17 [50.85] |
| Post-treatment* | Amitriptyline (25 mg) | 0.08 [0.57] | 1 | 365 | 29.06 [206.24] |
|  | Total (per year) | | | | 29.06 [206.24] |
| Diarrhea | Loperamide (2 mg) | 0.04 [0.26] | 8 | 3 | 0.87 [6.15] |
|  | IV fluids | 0.002 [0.01] | 2 | 3 | 0.01 [0.07] |
|  | Hospitalization^¥14^ | 368.21 [2,613.00] |  | 3 | 1,104.62 [7,839.00] |
|  | Total (per treatment course) | | | | 1,105.49 [7,845.22] |
| Febrile neutropenia | IV Ertapenem | 7.30 [51.82] | 1 | 3 | 21.90 [155.45] |
|  | Levofloxacin (500 mg) | 2.37 [16.83] | 1 | 4 | 9.49 [67.32] |
|  | SC Filgrastim (380 mcg) | 439.81 [3,121.13] | 1 | 3 | 1,319.42 [9,363.39] |
|  | IV fluids | 0.002 [0.01] | 2 | 3 | 0.01 [0.07] |
|  | Hospitalization^¥14^ | 368.21 [2,613.00] |  | 3 | 1,104.62 [7,839.00] |
|  | Total (per treatment course) | | | | 2,455.44 [17,425.23] |
| Nausea and Vomiting | IV Ondansetron | 0.59 [4.19] | 3 | 3 | 5.31 [37.71] |
|  | IV fluids | 0.002 [0.01] | 2 | 3 | 0.01 [0.07] |
|  | Hospitalization^¥14^ | 368.21 [2,613.00] |  | 3 | 1,104.62 [7,839.00] |
|  | Total (per treatment course) | | | | 1,109.94 [7,876.78] |
| Mucositis | Liquid oral lidocaine | 0.80 [5.64] | 3 | 5 | 11.93 [84.66] |
|  | IV fluids | 0.002 [0.01] | 1 | 5 | 0.01 [0.07] |
|  | Hospitalization^¥14^ | 368.21 [2,613.00] |  | 3 | 1,104.62 [7,839.00] |
|  | Total (per treatment course) | | | | 1,116.55 [7,923.72] |
| Hand-foot syndrome | Emla 5% cream | 1.30 [9.24] | 0 | 0 | 1.30 [9.24] |
|  | IV fluids | 0.002 [0.01] | 1 | 5 | 0.01 [0.07] |
|  | IV Morphine (10 mg) | 0.94 [6.67] | 4 | 3 | 11.30 [80.04] |
|  | Hospitalization^¥14^ | 368.21 [2,613.00] |  | 3 | 1,104.62 [7,839.00] |
|  | Total (per treatment course) | | | | 1,117.21 [7,928.34] |

I$=international dollars (2021). ZAR=South African rand (2021). IV=intravenous. SC=subcutaneous.

*****Grade 2/3 residual neuropathy.

^¥^Hospitalization costs assume admission to a general ward and include cost for general practitioner, specialized practitioner and facility fees.

**Supplementary Table 4. Direct costs associated with colon cancer surveillance following surgical resection.**^15,18^

| Procedure | Surveillance intervals,  months since surgery (frequency) | Cost component | Cost, I$ [ZAR] |
| --- | --- | --- | --- |
| Bloodwork | 0-12 months (every 3 months)  12-36 months (every 6 months)  36-60 months (every 12 months) | Drawing of blood | 6.06 [43.00] |
|  |  | Total (per procedure) | 6.06 [43.00] |
| Computed tomography scan* | 0-36 months (every 12 months) | Facility | 89.06 [632.00] |
|  |  | Personnel | 50.17 [356.00] |
|  |  | Radiology specialist | 153.88 [1,092.00] |
|  |  | Total (per procedure) | 293.10 [2,080.00] |
| Total fibre-optic colonoscopy | 12 months (once)  48 months (once) | Facility | 86.80 [616.00] |
|  |  | Gastroenterology specialist | 83.98 [596.00] |
|  |  | Anesthesia specialist | 93.14 [661.00] |
|  |  | Total (per procedure) | 263.93 [1,873.00] |

I$=international dollars (2021). ZAR=South African rand (2021).

*One computed tomography (CT) scan is also applied at 6 months after recurrence for patients receiving no adjuvant chemotherapy.

**Supplementary Table 5. External validation of model overall survival estimates (in 12-month increments). Overall survival model predictions by stage are compared to data from the CRCSA study, a prospective cohort study conducted in 5 hospitals in Johannesburg, ZA from 2016-2019**^19^**, which was not used for model development.**

| **Stage II** | | | | | | | | | |
| --- | --- | --- | --- | --- | --- | --- | --- | --- | --- |
| **CRCSA Data Estimate** | | | | | | **Model Prediction** | | | |
| **Time (mo)** | **N.risk** | **N.event** | **Survival** | **Lower 95%** | **Upper 95%** | **No treatment** | **6 mo cape** | **3 mo CAPOX** | **6 mo CAPOX** |
| 0 | 55 | 0 | 1 | - | - | 1 | 1 | 1 | 1 |
| 12 | 50 | 4 | 0.9269 | 0.8605 | 0.9985 | 0.94 | 0.944 | 0.950 | 0.951 |
| 24 | 50 | 4 | 0.9269 | 0.8605 | 0.9985 | 0.865 | 0.874 | 0.893 | 0.895 |
| 36 | 46 | 7 | 0.8705 | 0.7854 | 0.9649 | 0.796 | 0.809 | 0.839 | 0.842 |
| 48 | 30 | 11 | 0.7813 | 0.6734 | 0.9066 | 0.744 | 0.77 | 0.801 | 0.804 |
| 54 (Median FU) | 28 | 11 | 0.7813 | 0.6734 | 0.9066 | 0.728 | 0.757 | 0.792 | 0.795 |
| **Stage III** | | | | | | | | | |
| **CRCSA Data Estimate** | | | | | | **Model Prediction** | | | |
| **Time (mo)** | **N.risk** | **N.event** | **Survival** | **Lower 95%** | **Upper 95%** | **No treatment** | **6 mo cape** | **3 mo CAPOX** | **6 mo CAPOX** |
| 0 | 79 | 0 | 1 | - | - | 1 | 1 | 1 | 1 |
| 12 | 68 | 11 | 0.8593 | 0.7856 | 0.94 | 0.878 | 0.927 | 0.936 | 0.936 |
| 24 | 58 | 19 | 0.754 | 0.664 | 0.857 | 0.685 | 0.779 | 0.827 | 0.812 |
| 36 | 49 | 26 | 0.6611 | 0.5631 | 0.7763 | 0.58 | 0.687 | 0.752 | 0.739 |
| 41 (Median FU) | 41 | 29 | 0.6207 | 0.5206 | 0.7399 | 0.543 | 0.66 | 0.734 | 0.721 |

mo = months. CAPOX=capecitabine and oxaliplatin. cape = capecitabine.

**Supplementary Table 6. Model inputs in the subgroup analysis for South African a) high-risk stage II and b) stage III colon cancer adjuvant chemotherapy simulation models: monthly colon cancer recurrence probabilities post-surgery.**

**6a)**

| High-Risk Stage II^20^ | | | | |
| --- | --- | --- | --- | --- |
| Strategy | T4 stage | T3 stage | Less than 10 lymph nodes evaluated | 10 or more lymph nodes evaluated |
| CAPOX 3 months^3^ | 0.0003-0.0081 | 0.0002-0.0030 | 0.0002-0.0046 | 0.0002-0.0022 |
| CAPOX 6 months^3^ | 0.0003-0.0078 | 0.0002-0.0029 | 0.0002-0.0044 | 0.0002-0.0021 |
| Capecitabine 6 months^2^ | 0.0017-0.0110 | 0.0011-0.0040 | 0.0012-0.0062 | 0.0001-0.0029 |
| No Adjuvant Chemotherapy^1^ | 0.0024-0.0121 | 0.0016-0.0044 | 0.0017-0.0069 | 0.0016-0.0032 |

**6b)**

| Stage III | | |
| --- | --- | --- |
| Strategy | High risk for colon cancer recurrence | Low risk for colon cancer recurrence |
| CAPOX 3 months^6^ | 0.0016-0.0202 | 0.0002-0.0036 |
| CAPOX 6 months^6^ | 0.0006-0.0171 | 0.0005-0.0044 |
| No Adjuvant Chemotherapy^21^ | 0.0030-0.0320 | 0.0020-0.0200 |

CAPOX=capecitabine and oxaliplatin.

**Supplementary Table 7. Cost-effectiveness results from the South African high-risk stage II colon cancer adjuvant chemotherapy T staging subgroup simulation models, with no adjuvant chemotherapy as comparator and a willingness-to-pay (WTP) threshold equal to South Africa’s 2021 GDP per capita.**

| Strategy | Total Cost, I$ [ZAR] | Overall Survival,  years | DALYs averted | ICER,  I$/DALY averted | NMB, I$ [ZAR] |
| --- | --- | --- | --- | --- | --- |
| High-risk stage II T4 stage | | | | | |
| No Adjuvant Chemotherapy | 2,852 [20,240] | 10.52 | 0.00 | -- | -2,827 [-20,061] |
| Capecitabine 6 months | 3,622 [25,702] | 11.54 | 1.37 | extended dominated | 15,183 [107,751] |
| CAPOX 3 months | 4,559 [32,355] | 13.01 | 3.36 | 508 | 41,744 [296,257] |
| CAPOX 6 months | 5,841 [41,453] | 13.11 | 3.50 | 853 | 42,400 [300,910] |
| High-risk stage II T3 stage | | | | | |
| No Adjuvant Chemotherapy | 2,672 [18,963] | 14.11 | 0.00 | -- | -2,700 [-19,162] |
| Capecitabine 6 months | 3,764 [26,714] | 14.70 | 0.79 | extended dominated | 7,098 [50,373] |
| CAPOX 3 months | 4,021 [28,536] | 15.63 | 2.04 | 659 | 24,122 [171,193] |
| CAPOX 6 months | 5,341 [37,905] | 15.68 | 2.11 | 1,266 | 23,646 [167,818] |

Overall survival is presented as life years, undiscounted and unadjusted for disability. All other results have a global annual discounting of 5% (r=0.05) applied to all costs and effectiveness calculations and are adjusted using annual disability weights. GDP=gross domestic product. I$=international dollars (2021). ZAR=South African rand (2021). DALY=disability-adjusted life-year. ICER=incremental cost-effectiveness ratio. NMB=net monetary benefit. CAPOX=capecitabine and oxaliplatin.

**Supplementary Table 8. Cost-effectiveness results from the South African high-risk stage II colon cancer adjuvant chemotherapy lymph node evaluation subgroup simulation models, with no adjuvant chemotherapy as comparator and a willingness-to-pay (WTP) threshold equal to South Africa’s 2021 GDP per capita.**

| Strategy | Total Cost, I$ [ZAR] | Overall Survival,  years | DALYs averted | ICER,  I$/DALY averted | NMB, I$ [ZAR] |
| --- | --- | --- | --- | --- | --- |
| High-risk stage II less than 10 lymph nodes evaluated | | | | | |
| No Adjuvant Chemotherapy | 2,729 [19,367] | 12.92 | 0.00 | -- | -2,763 [-19,607] |
| Capecitabine 6 months | 3,716 [26,371] | 13.67 | 1.00 | extended dominated | 10,066 [71,436] |
| CAPOX 3 months | 4,201 [29,813] | 14.76 | 2.47 | 595 | 29,804 [211,522] |
| CAPOX 6 months | 5,508 [39,091] | 14.83 | 2.56 | 1,084 | 29,740 [211,066] |
| High-risk stage II 10 or more lymph nodes evaluated | | | | | |
| No Adjuvant Chemotherapy | 2,648 [18,790] | 14.69 | 0.00 | -- | -2,676 [-18,989] |
| Capecitabine 6 months | 3,790 [26,899] | 15.20 | 0.68 | extended dominated | 5,579 [39,596] |
| CAPOX 3 months | 3,928 [27,881] | 16.08 | 1.87 | 686 | 21,743 [154,311] |
| CAPOX 6 months | 5,255 [37,297] | 16.12 | 1.91 | 1,363 | 21,041 [149,331] |

Overall survival is presented as life years, undiscounted and unadjusted for disability. All other results have a global annual discounting of 5% (r=0.05) applied to all costs and effectiveness calculations and are adjusted using annual disability weights. GDP=gross domestic product. I$=international dollars (2021). ZAR=South African rand (2021). DALY=disability-adjusted life-year. ICER=incremental cost-effectiveness ratio. NMB=net monetary benefit. CAPOX=capecitabine and oxaliplatin.

**Supplementary Table 9. Cost-effectiveness results from the South African stage III colon cancer adjuvant chemotherapy risk-stratified subgroup simulation models, with no adjuvant chemotherapy as comparator and a willingness-to-pay (WTP) threshold equal to South Africa’s 2021 GDP per capita.**

| Strategy | Total Cost, I$ [ZAR] | Overall Survival,  years | DALYs averted | ICER,  I$/DALY averted | NMB, I$ [ZAR] |
| --- | --- | --- | --- | --- | --- |
| Low-risk stage III | | | | | |
| CAPOX 3 months | 5,007 [39,906] | 14.11 | 7.37 | -- | 96,422 [768,480] |
| No Adjuvant Chemotherapy | 5,217 [41,581] | 8.73 | 0.00 | dominated | -5,277 [-42,061] |
| CAPOX 6 months | 6,368 [50,754] | 13.89 | 7.06 | dominated | 90,751 [723,286] |
| High-risk stage III | | | | | |
| CAPOX 3 months | 5,949 [47,412] | 10.94 | 6.71 | -- | 86,433 [688,868] |
| No Adjuvant Chemotherapy | 5,992 [47,758] | 6.05 | 0.00 | dominated | -5,937 [-47,315] |
| CAPOX 6 months | 6,984 [55,663] | 11.43 | 7.36 | 1,587 | 94,374 [752,164] |

Overall survival is presented as life years, undiscounted and unadjusted for disability. All other results have a global annual discounting of 5% (r=0.05) applied to all costs and effectiveness calculations and are adjusted using annual disability weights. GDP=gross domestic product. I$=international dollars (2021). ZAR=South African rand (2021). DALY=disability-adjusted life-year. ICER=incremental cost-effectiveness ratio. NMB=net monetary benefit. CAPOX=capecitabine and oxaliplatin.

**Supplementary Table 10. Cost-effectiveness results from the scenario analysis for high-risk stage II and stage III colon cancer for XELIRI for metastatic treatment, with no adjuvant chemotherapy as comparator and a willingness-to-pay (WTP) threshold equal to South Africa’s 2021 GDP per capita.**

| Strategy | Total Cost, I$ [ZAR] | Overall Survival,  years | DALYs averted | ICER,  I$/DALY averted | NMB, I$ [ZAR] |
| --- | --- | --- | --- | --- | --- |
| High-risk stage II | | | | | |
| No Adjuvant Chemotherapy | 2,513 [17,832] | 13.69 | 0.00 | -- | -2,512 [-17,830] |
| CAPOX 3 months | 3,586 [25,446] | 15.33 | 2.21 | 486 | 26,816 [190,314] |
| Capecitabine 6 months | 3,748 [26,599] | 14.34 | 0.87 | dominated | 8,221 [58,342] |
| CAPOX 6 months | 4,914 [34,873] | 15.39 | 2.28 | 1,053 | 26,471 [187,865] |
| Stage III | | | | | |
| No Adjuvant Chemotherapy | 3,506 [24,882] | 9.00 | 0.00 | -- | -3,535 [-25,086] |
| Capecitabine 6 months | 3,737 [26,522] | 11.87 | 3.98 | extended dominated | 51,096 [362,628] |
| CAPOX 3 months | 3,745 [26,581] | 12.99 | 5.46 | 44 | 71,453 [507,105] |
| CAPOX 6 months | 5,047 [35,821] | 12.79 | 5.19 | dominated | 66,455 [471,630] |

Overall survival is presented as life years, undiscounted and unadjusted for disability. All other results have a global annual discounting of 5% (r=0.05) applied to all costs and effectiveness calculations and are adjusted using annual disability weights. GDP=gross domestic product. I$=international dollars (2021). ZAR=South African rand (2021). DALY=disability-adjusted life-year. ICER=incremental cost-effectiveness ratio. NMB=net monetary benefit. CAPOX=capecitabine and oxaliplatin.

**Supplementary Figure 1. Systemic treatment regimens at metastatic colon cancer recurrence for the a) base case and b) scenario analyses.**

**
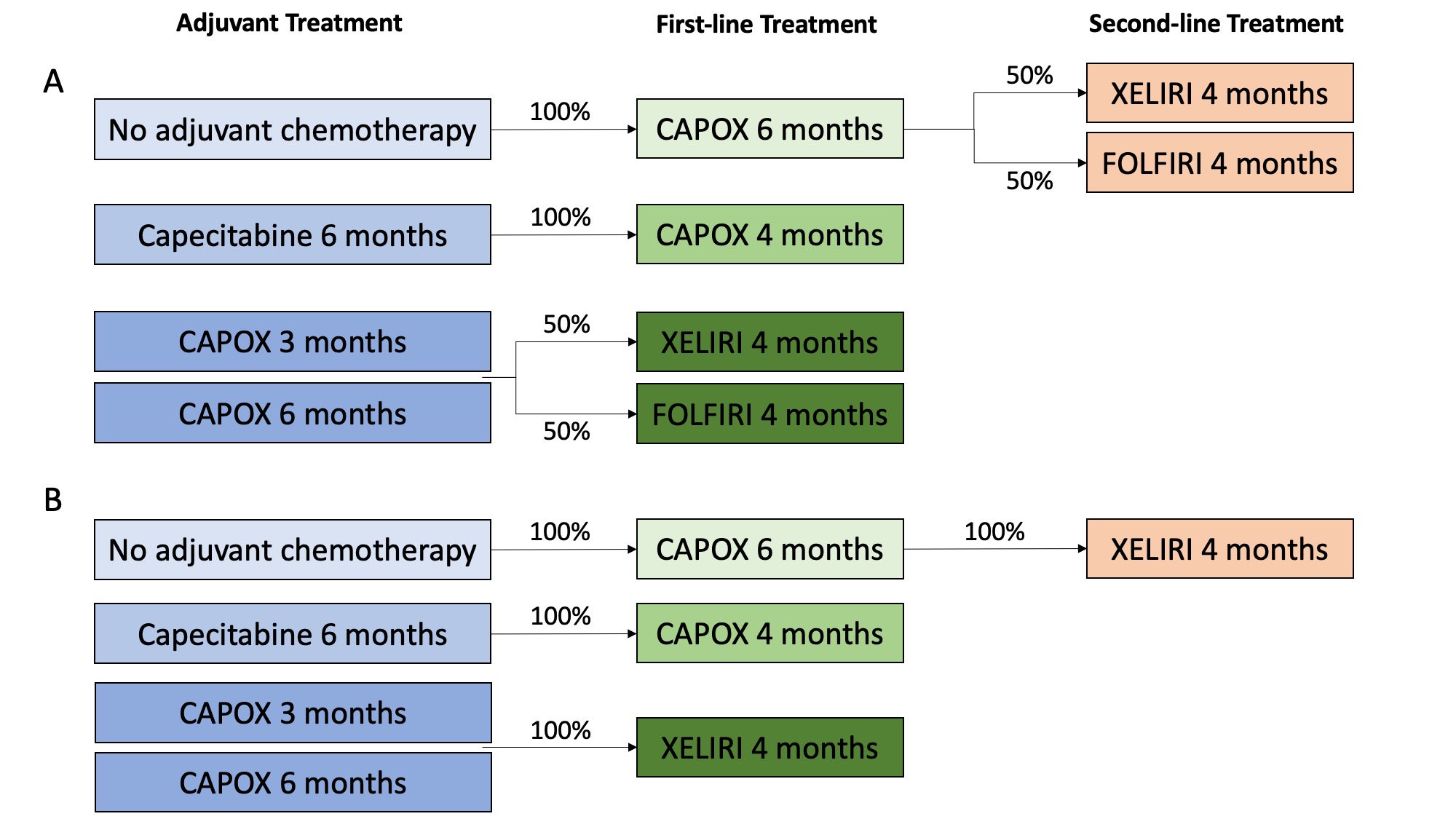
**

CAPOX=capecitabine and oxaliplatin. XELIRI=capecitabine and irinotecan. FOLFIRI=fluorouracil, leucovorin, and irinotecan.

**Supplementary Figure 2. Comparison of clinical trial data (grey dotted line) and model outputs using US background death (orange line) and ZA background death (blue line) for each treatment arm for high-risk stage II colon cancer.**

**
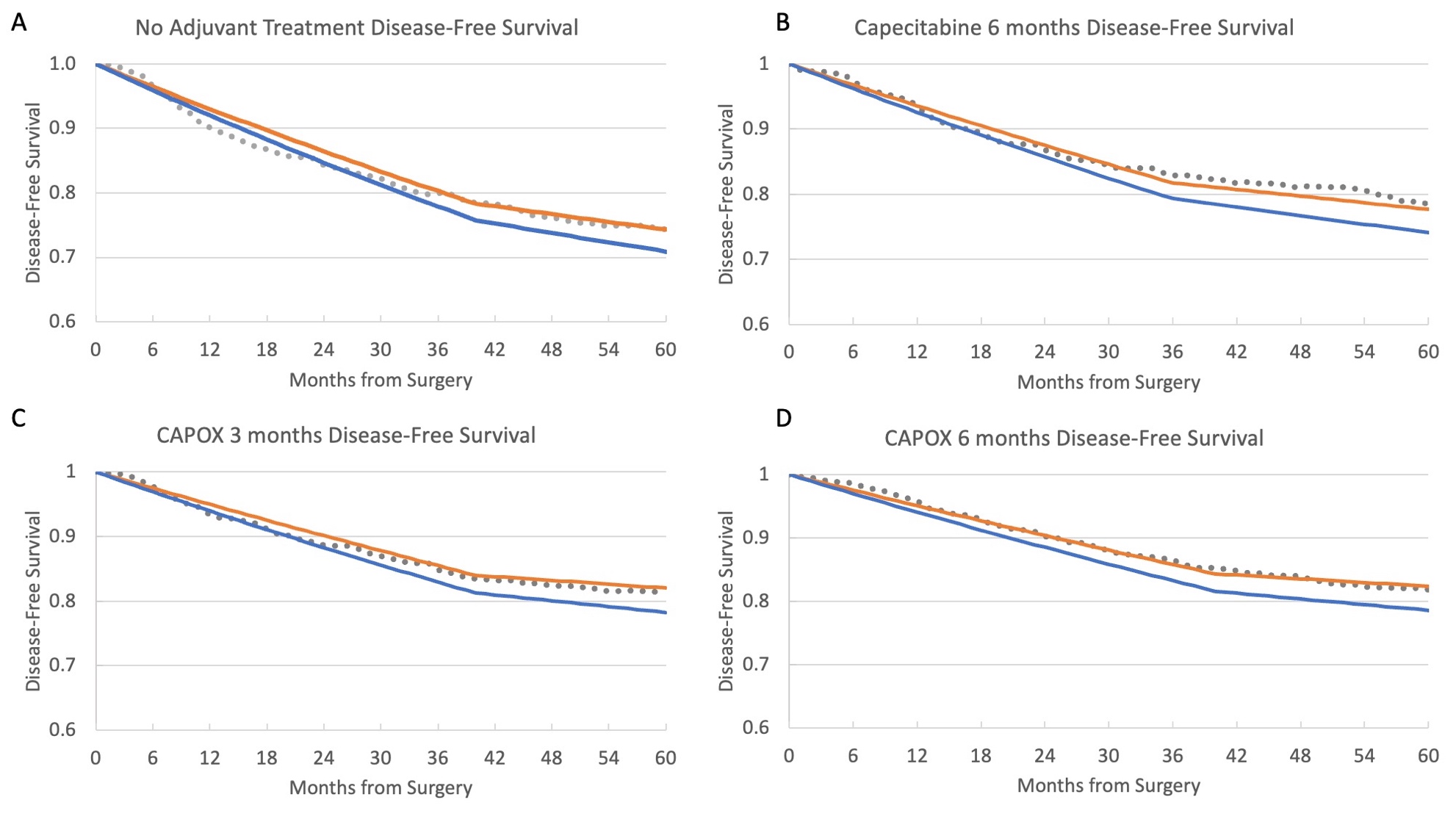
**

The model was populated with recurrence probabilities from published RCTs done in the US. Model DFS outputs using a background death rate representative of the study population (US) were within 1.5% of the published rates. In the final model, South African background death rates were used in replacement of the US background death to contextualize our analysis to ZA, which resulted in lower DFS estimates than those in the published RCTs, as expected. US=United States. ZA=South Africa. DFS=Disease-Free Survival. RCT=randomized controlled clinical trial. CAPOX=capecitabine and oxaliplatin.

**Supplementary Figure 3. Comparison of clinical trial data (grey dotted line) and model outputs using US background death (orange line) and ZA background death (blue line) for each treatment arm for stage III colon cancer.**

**
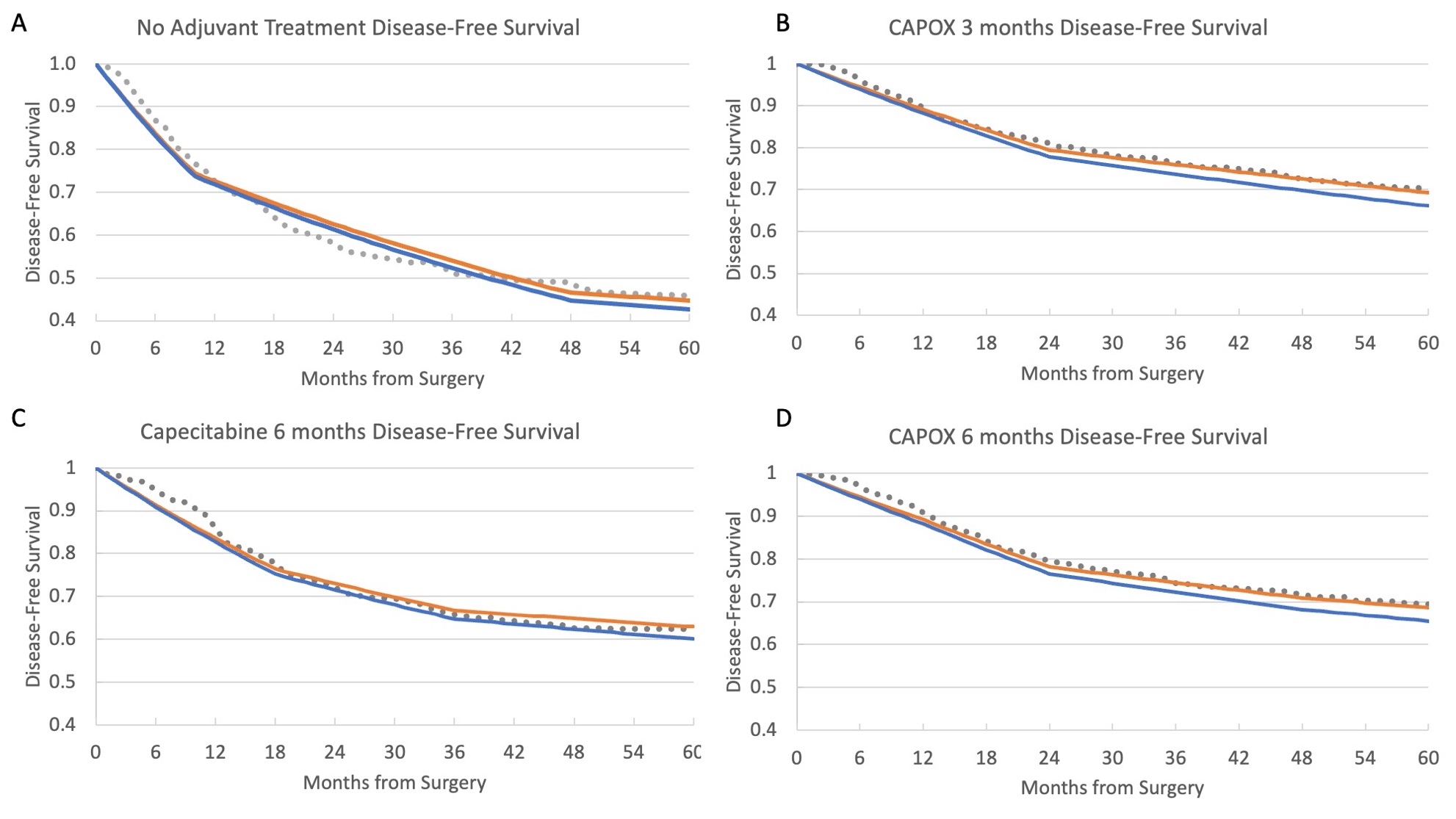
**

The model was populated with recurrence probabilities from published RCTs done in the US. Model DFS outputs using a background death rate representative of the study population (US) were within 1.5% of the published rates. In the final model, South African background death rates were used in replacement of the US background death to contextualize our analysis to ZA, which resulted in lower DFS estimates than those in the published RCTs, as expected. US=United States. ZA=South Africa. DFS=Disease-Free Survival. RCT=randomized controlled clinical trial. CAPOX=capecitabine and oxaliplatin.

**Supplementary Figure 4. Probabilistic sensitivity analysis (PSA) scatterplot of the results from the South African a) high-risk stage II and b) stage III colon cancer adjuvant chemotherapy simulation model (base case scenario).**


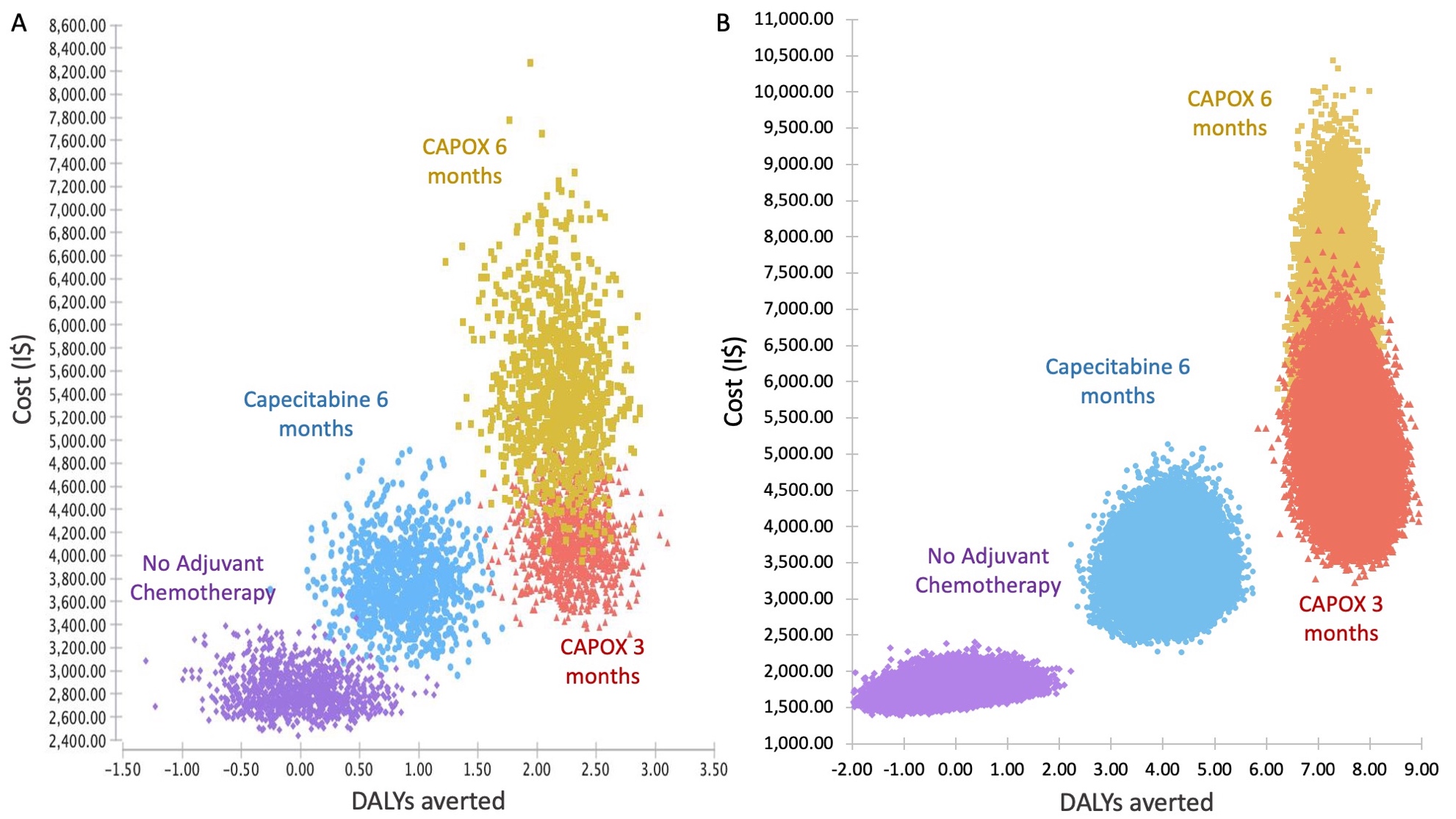


The PSA was conducted for 100,000 random samples, with a WTP threshold equivalent to the 2021 GDP per capita in South Africa. I$=international dollars (2021). DALY=disability-adjusted life-year. CAPOX=capecitabine and oxaliplatin. WTP=Willingness-to-pay. GDP=gross domestic product.

**Supplementary Figure 5. Probabilistic sensitivity analysis (PSA) cost-effectiveness acceptability curve of the results from the South African a) high-risk stage II and b) stage III colon cancer adjuvant chemotherapy simulation model from 0.5 times to 3 times the willingness-to-pay (WTP) threshold.**

**
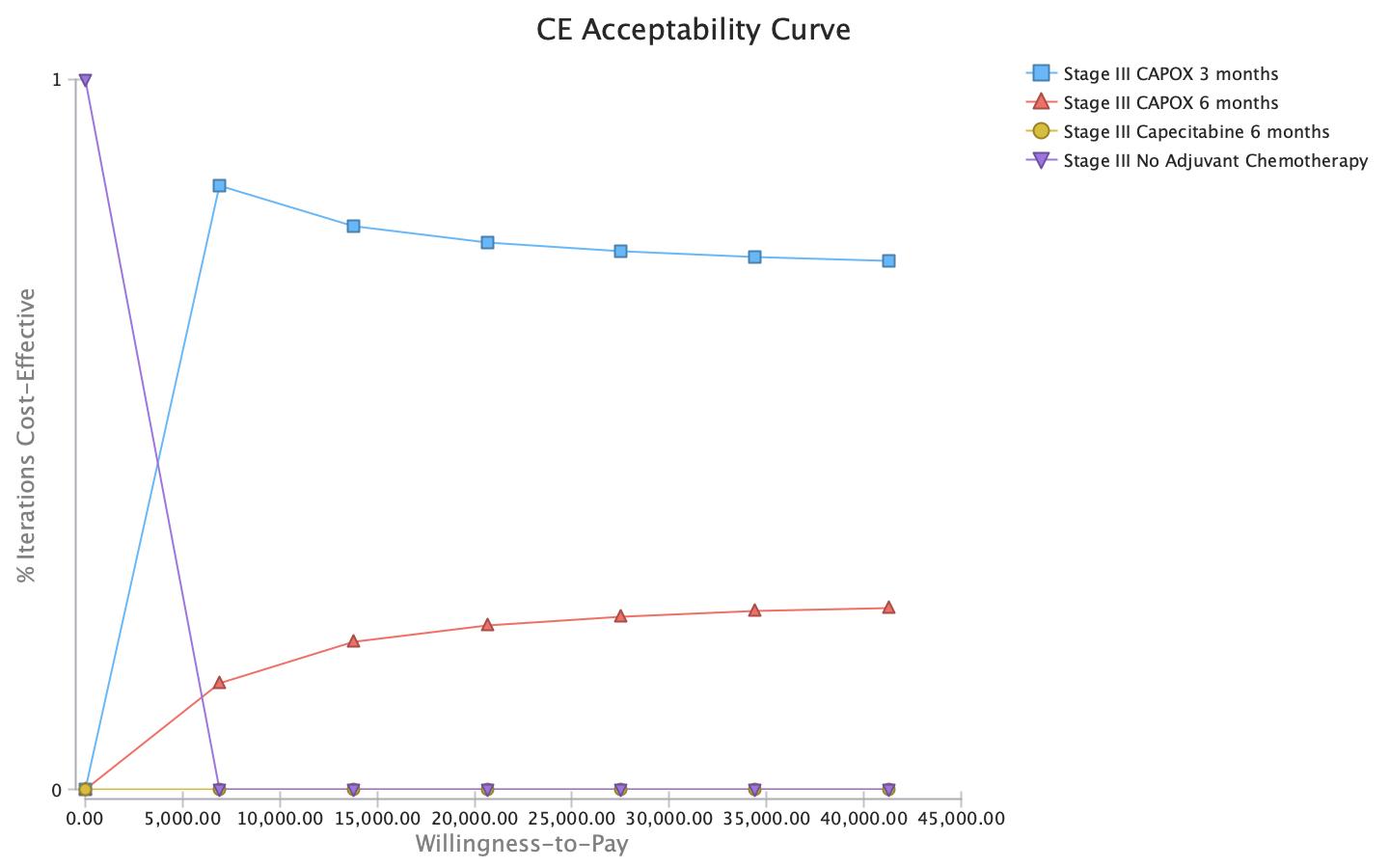

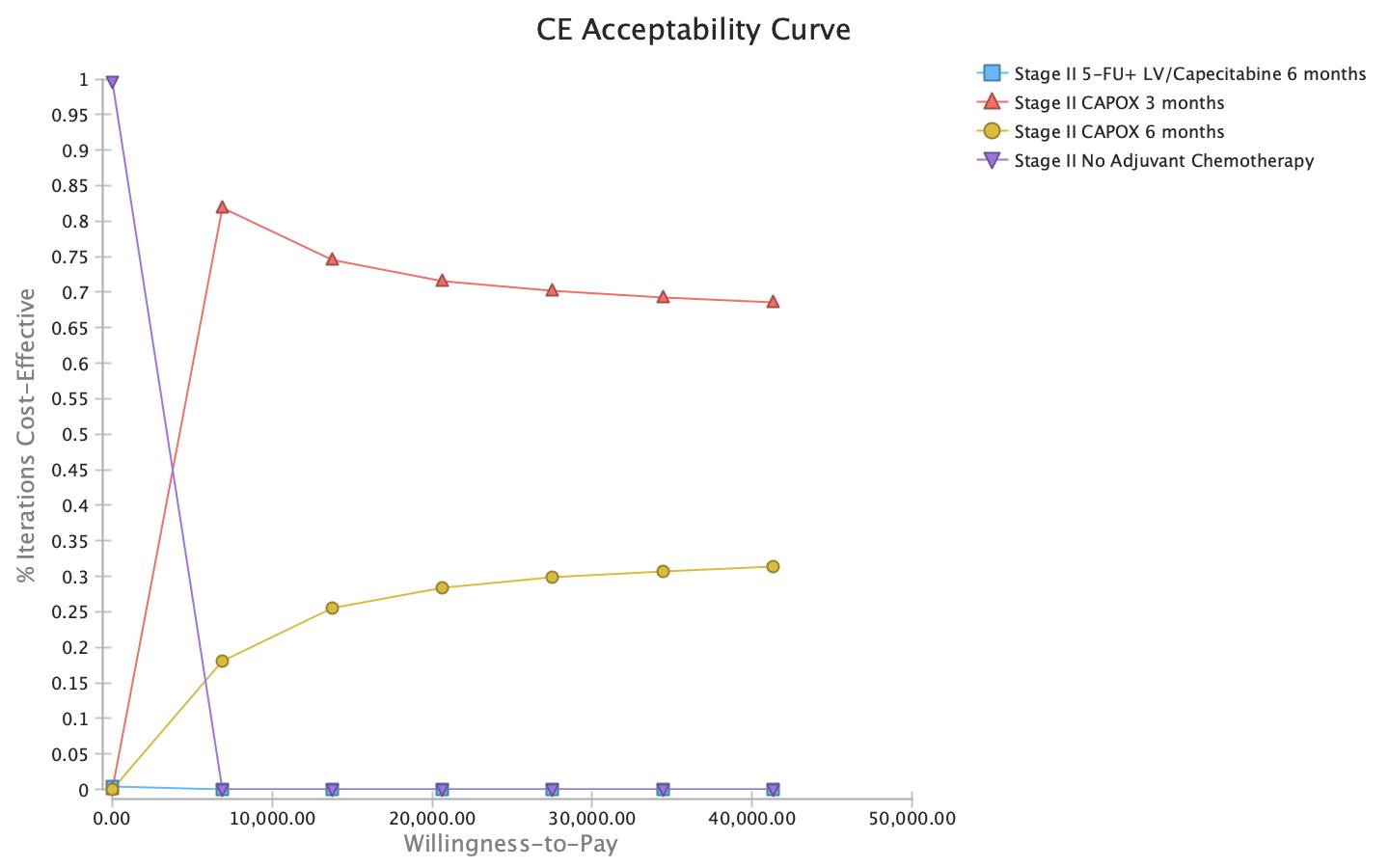
**

Stage III CAPOX 3 months

Stage III CAPOX 6 months

Stage III Capecitabine 6 months

Stage III No adjuvant chemotherapy

High-risk stage II Capecitabine 6 months

High-risk stage II CAPOX 3 months

High-risk stage II CAPOX 6 months

High-risk stage II No adjuvant chemotherapy

**
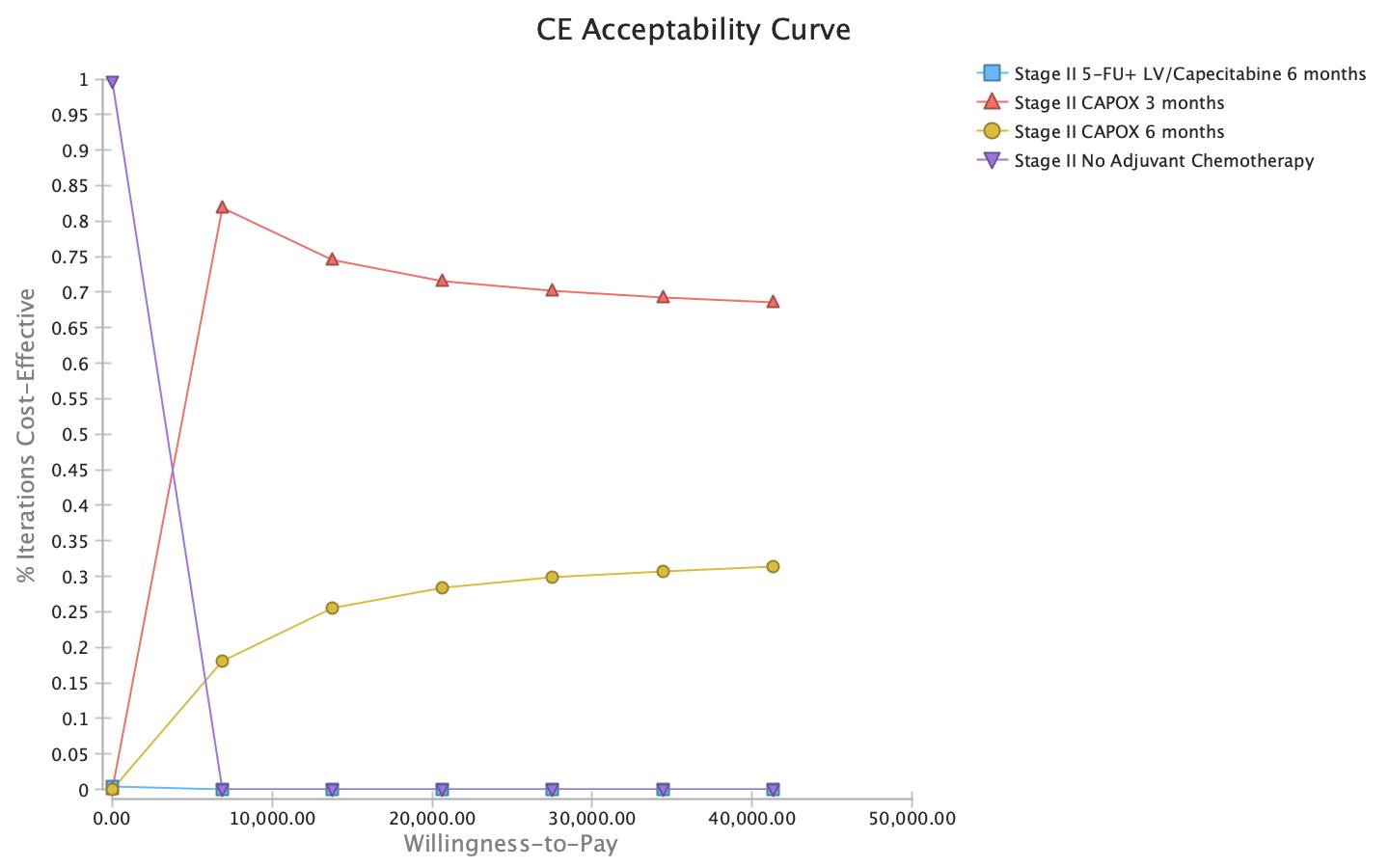

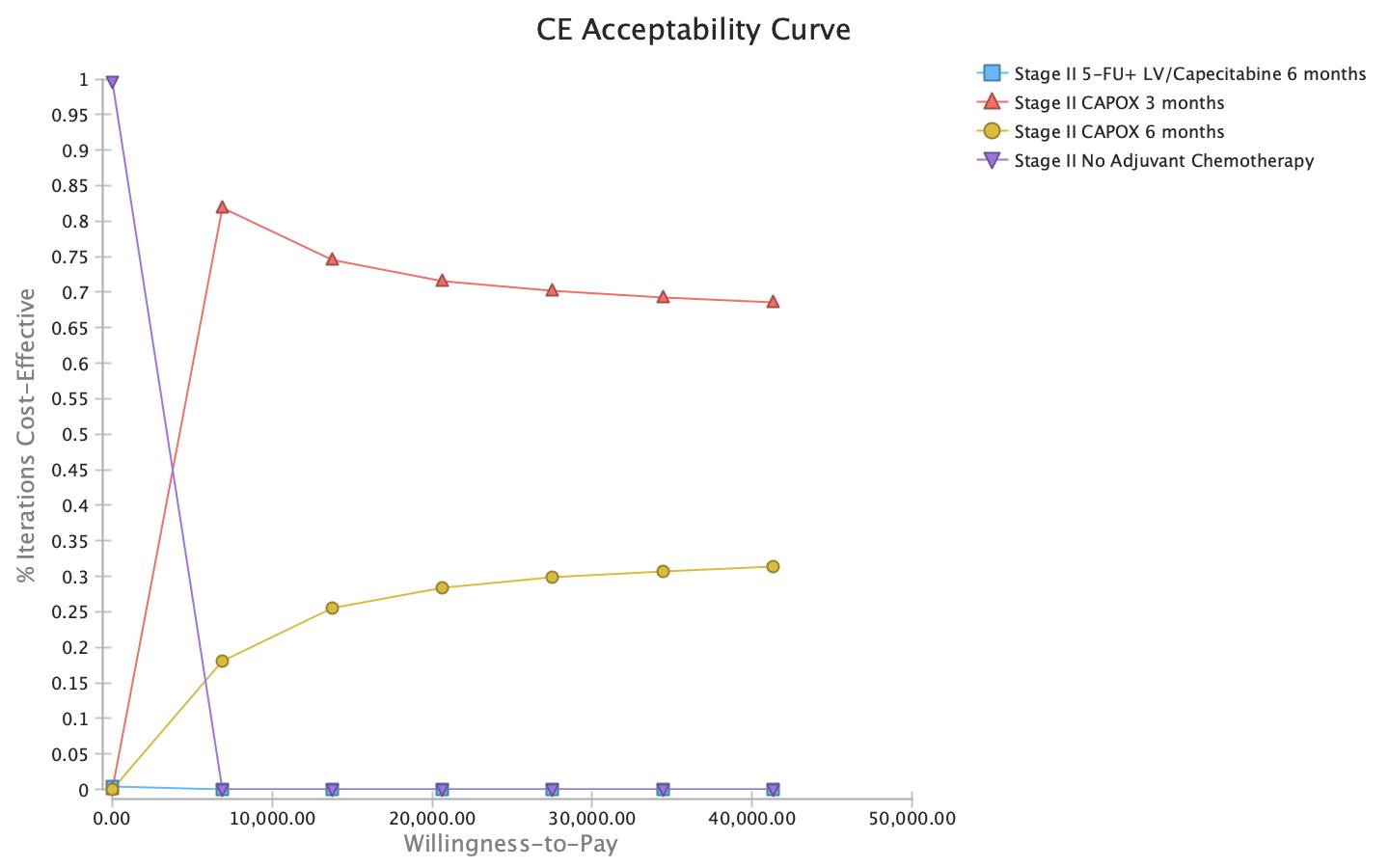
**

The PSA was conducted for 100,000 random samples, with a WTP range from one half to three times the 2021 GDP per capita in South Africa (I$), used as the base case WTP threshold. I$=international dollars (2021). CAPOX=capecitabine and oxaliplatin. WTP=Willingness-to-pay. GDP=gross domestic product.

**References**

1. Kumar A, Kennecke HF, Renouf DJ, et al. Adjuvant chemotherapy use and outcomes of patients with high-risk versus low-risk stage II colon cancer. *Cancer*. 2015;121(4):527-534. doi:10.1002/cncr.29072

2. Tournigand C, André T, Bonnetain F, et al. Adjuvant Therapy With Fluorouracil and Oxaliplatin in Stage II and Elderly Patients (between ages 70 and 75 years) With Colon Cancer: Subgroup Analyses of the Multicenter International Study of Oxaliplatin, Fluorouracil, and Leucovorin in the Adjuvant Treatment of Colon Cancer Trial. *JCO*. 2012;30(27):3353-3360. doi:10.1200/JCO.2012.42.5645

3. Iveson TJ, Sobrero AF, Yoshino T, et al. Duration of Adjuvant Doublet Chemotherapy (3 or 6 months) in Patients With High-Risk Stage II Colorectal Cancer. *JCO*. 2021;39(6):631-641. doi:10.1200/JCO.20.01330

4. Moertel CG. Fluorouracil plus Levamisole as Effective Adjuvant Therapy after Resection of Stage III Colon Carcinoma: A Final Report. *Ann Intern Med*. 1995;122(5):321. doi:10.7326/0003-4819-122-5-199503010-00001

5. Twelves C, Wong A, Nowacki MP, et al. Capecitabine as Adjuvant Treatment for Stage III Colon Cancer. *New England Journal of Medicine*. 2005;352(26):2696-2704. doi:10.1056/NEJMoa043116

6. André T, Meyerhardt J, Iveson T, et al. Effect of duration of adjuvant chemotherapy for patients with stage III colon cancer (IDEA collaboration): final results from a prospective, pooled analysis of six randomised, phase 3 trials. *The Lancet Oncology*. 2020;21(12):1620-1629. doi:10.1016/S1470-2045(20)30527-1

7. Sobrero AF, Andre T, Meyerhardt JA, et al. Overall survival (OS) and long-term disease-free survival (DFS) of three versus six months of adjuvant (adj) oxaliplatin and fluoropyrimidine-based therapy for patients (pts) with stage III colon cancer (CC): Final results from the IDEA (International Duration Evaluation of Adj chemotherapy) collaboration. *JCO*. 2020;38(15_suppl):4004-4004. doi:10.1200/JCO.2020.38.15_suppl.4004

8. Database of Medicine Prices 24 December 2021. Published online December 24, 2021. Accessed January 24, 2022. http://www.mpr.gov.za/

9. HP04-2020ONC: Supply and Delivery of Oncology and Immunological Agents to the Department of Health for Period 1 July 2020 to 30 June 2022. Published online May 14, 2020. http://health.gov.za/tender%20archives/docs/contracts/2020/HP04-2020ONC_Contract_Circular_14_May_2020.pdf

10. Steel G, Ruff P, Dreosti L. Improving Access to Oncology Treatments in a Resource Constrained Setting Using Pharmacoeconomic Analysis. In: ; 2011.

11. Gauteng Department of Health. Health: Republic of South Africa. Published August 14, 2021. Accessed January 24, 2022. https://www.govpage.co.za/gauteng-health-vacancies-blog/gauteng-department-of-health4943873

12. Herbst C lee, Miot JK, Moch SL, Ruff P. Access to colorectal cancer (CRC) chemotherapy and the associated costs in a South African public healthcare patient cohort. *Journal of Cancer Policy*. 2018;15:18-24. doi:10.1016/j.jcpo.2017.11.005

13. Statistical Release P0141: Consumer Price Index March 2021. Published online May 19, 2021. http://www.statssa.gov.za/publications/P0141/P0141March2021.pdf

14. UPFS Tariff Committee. Uniform Patient Fee Schedule 2021 - Annexure A2. Published online April 1, 2021. Accessed January 21, 2022. http://www.health.gov.za/uniform-patient-fee-schedule/

15. Full-Paying Patients. Published online May 30, 2018. Accessed January 19, 2021. https://www.westerncape.gov.za/dept/health/documents/public_in/public_info

16. Grothey A, Sobrero AF, Shields AF, et al. Duration of Adjuvant Chemotherapy for Stage III Colon Cancer. *N Engl J Med*. 2018;378(13):1177-1188. doi:10.1056/NEJMoa1713709

17. Xu RH, Muro K, Morita S, et al. Modified XELIRI (capecitabine plus irinotecan) versus FOLFIRI (leucovorin, fluorouracil, and irinotecan), both either with or without bevacizumab, as second-line therapy for metastatic colorectal cancer (AXEPT): a multicentre, open-label, randomised, non-inferiority, phase 3 trial. *The Lancet Oncology*. 2018;19(5):660-671. doi:10.1016/S1470-2045(18)30140-2

18. UPFS Tariff Committee. Uniform Patient Fee Schedule 2021 - Annexure A1. Published online April 1, 2021. Accessed January 27, 2022. http://www.health.gov.za/uniform-patient-fee-schedule/

19. Bebington B, Singh E, Fabian J, et al. Design and methodology of a study on colorectal cancer in Johannesburg, South Africa. *JGH Open*. 2018;2(4):139-143. doi:10.1002/jgh3.12061

20. Jongeneel G, Greuter MJE, van Erning FN, et al. Modeling Personalized Adjuvant TreaTment in EaRly stage coloN cancer (PATTERN). *Eur J Health Econ*. 2020;21(7):1059-1073. doi:10.1007/s10198-020-01199-4

21. Wilkinson NW, Yothers G, Lopa S, Costantino JP, Petrelli NJ, Wolmark N. Long-Term Survival Results of Surgery Alone Versus Surgery Plus 5-Fluorouracil and Leucovorin for Stage II and Stage III Colon Cancer: Pooled Analysis of NSABP C-01 Through C-05. A Baseline from Which to Compare Modern Adjuvant Trials. *Ann Surg Oncol*. 2010;17(4):959-966. doi:10.1245/s10434-009-0881-y
